# Evaluation of in vitro-geranium (*Pelargonium graveolens*) plants affected by irradiation and chemical mutagens

**DOI:** 10.1186/s12870-025-07170-w

**Published:** 2025-10-23

**Authors:** Omneya F. Abou El-Leel, Eman F. AbouEl-Leil, Amina A. Aly

**Affiliations:** 1 Medicinal and Aromatic Plants Research Department, Horticulture Research Institute (HRI), Agricultural Research Centre (ARC), Cairo, Egypt; 2 Natural Products Research Department, National Centre for Radiation Research and Technology, Egyptian Atomic Energy Authority (EAEA), Cairo, Egypt

**Keywords:** Volatile oil, RAPD, ISSR, Mutant, Geranium (*Pelargonium graveolens*)

## Abstract

**Background:**

The current investigation was performed to establish the influence of gamma rays, He–Ne laser and colchicine & dinitroaniline as chemical mutagens on micropropagation of geranium (*Pelargonium graveolens*), its oil components and genetic diversity. Survival percentage, plant height, branch numbers, leaf numbers, fresh weight and essential oil percentage were recorded.

**Results:**

Applications of gamma rays and He–Ne laser light had a vital impact on vegetative development characters and oil induction of geranium. The treated samples also showed improving the volatile oil and their compounds when compared to the mother plant. The highest result of γ-rays different doses was confirmed on plant height, branch numbers, leaf numbers and volatile oil percent. Laser light samples exhibited a further pronounced outcome on the development parameters of *P. graveolens*. Colchicine and dinitroaniline increased survival percentages and other related potential agronomic traits. The GC-MS analyses of the volatile oil showed that the total identified components are ranged from 79.35% in mother plants to 96.97% in 5 Gy of γ-rays. Here, 44 volatile compounds included 5 important were identified in the essential oils; citronellol, geraniol, citronellylformate, L-linalool and l-menthone were most predominant of the all samples. At molecular level, 6 RAPD primers showed a sum of 60 amplified fragments, of which fifty (83.33%) were polymorphic fragment. The number of sum of total amplified fragments scored per primer ranged between 7 (primer-OP-Q18) to 16 (primer-OP-A01). Fourteen out of 60 RAPD fragments were found to be useful as specific markers. In ISSR analysis, 6 ISSR primers amplified variable banding pattern. A sum of 59 Out of 73 ISSR fragments, were polymorphic. Also, 27 produced fragments were considered as specific markers. In the recent investigation certain physical mutagens (γ-rays and laser beam) and chemical mutagens (colchicine and dinitroanilne) were performed for improving morpho-physiological and yield traits.

**Conclusion:**

The applied mutagens resulted in noteworthy variations in all assessed traits due to the usage of mutagens, indicating their efficiency for causing more useful genetic diversity in other plants material for improving agronomic features.

**Supplementary Information:**

The online version contains supplementary material available at 10.1186/s12870-025-07170-w.

## Background

Geranium (*Pelargonium graveolens*) is one of the most valuable export medicinal and aromatic plants in Egypt. It belongs to the Geraniaceae family, which is grown for its valuable oils, which are utilized in the cosmetics and food industries [1]. Geranium is native to southern Africa and is broadly grown in several countries, mainly in Russia, Egypt, Morocco, Algeria, Japan, and Congo, as well as several nations in Europe and Central America [2]. Bioactive natural plants products derived from *P. graveolens* extracts have been found to have a range of biological and pharmacological activities, including antioxidants [3], antibacterial [4], antifungal [5], hypoglycemic [6], anti-inflammatory [7], and anticancer [8]. These activities could be related to the presence of citronellol, linalool and geraniol as main bioactive components found in *P. graveolens* essential oil [9]. According to Jaradat et al. [10] on herbs and plant extracts may result in breakthrough treatment for key health problems like cancer, diabetes, obesity, oxidative stress, and antibiotic resistance. Citronellol, geraniol, and linalool, found in geranium oil, have the ability to relax smooth muscles. This makes it valuable oil for skincare as it effectively good in opening skin pores and cleaning oily complexions. It can be also utilized in reducing pain due to post-herpetic neuralgia and treating dysentery, hemorrhoids, inflammation, heavy menstrual flows and cancer [11]. Since geranium has been vegetatively propagated and does not produce seeds, it is predicted that the geranium has accumulated a large number of recessive mutations and a high degree of heterozygosity. Significant intra-clonal differences have been obtained in scented geranium plants grown from leave and roots suckers [12, 13]. Mutation breeding played a significant role which helped thousands of plant varieties to enhance their phenotypic traits like agronomic performance, nutrition qualities, disease resistance, and stress tolerance, to herbicides via applying physical and chemical mutagen agents to generate mutations, and that started from the beginning of the 20th century [14, 15]. Furthermore, novel methods for targeted mutagenesis in plants have been developed as a result of more recent advancements in plant breeding [16]. Ionizing radiation having low wavelength and a high penetrating power, γ-rays interacts with atoms or molecules to create free radicals inside the cells. Plant cell contents can be damaged or modified by free radicals [17]. According to Aly et al. [18], depending on the irradiation dose level, gamma radiation has been shown to impact plant shape, anatomy, physiochemical properties, and seed germination. These impacts include modifications in the plant cellular structure and metabolism, e.g., dilation of the thylakoid membrane, alteration in photosynthesis, modulation of the antioxidative system and accumulation of phenolic compounds [19, 20]. There are several different types of lasers, including cobalt laser (green) and argon laser (blue). One of those types is helium-neon laser (He-Ne), which emits red light at a wavelength of 632.8 nm in the portion of a milliwatt (mW). Laser irradiation offers a great deal of possible applications in photobiology. Due to these traits, laser irradiation is useful in both biological and agricultural domains, wherever it can have multiple effects on crop plants [21, 22]. Furthermore, Qiu et al. [23] discovered that the plants originated from He–Ne laser pretreated seeds have significantly higher levels of antioxidative enzymes, relative tissue water and malondialdehyde concentrations compared to untreated plants. The ability of laser-treated plants to down-regulate several miRNA species was found to be strongly associated with their higher performance. It has been observed that, in germinating seeds, increasing the irradiation intensity and exposure time of the He-Ne laser increases the contents of endogenous growth-promoting hormones with a concurrent decrease in the growth-inhibiting compounds [24]. As cited previously by Aslam et al. [25] that maize, barley, and wheat plants originating from laser-irradiated seeds had a substantially larger leaf surface area compared with those generated from un-irradiated seeds. Laser as biostimulator has emerged to be a promising optical technique to improve crops germination and growth [26]. The influence of laser for enhancing the growth traits of different crops has previously been established, like wheat [27, 28], pea [29], eggplant [30], soybean [31], and tomato [32]. Moreover, plants that receive pre-primed laser radiation induces DNA polymorphism in the plants that derive from it. This DNA polymorphism could be identified using ISSR markers [33]. Applying chemical agents for mutation induction in the plants genome to enhance crop yields. Several of these substances induce chromosomal aberration impacts in plants as a result of generating reactive free radicals [34].

An easy technique for improving germination response along with additional relevant prospective agricultural features in plants is to use chemical mutagenic substances [35]. Another mutagen that is employed for both causing mutations and the establishment of polyploidy in plant is colchicine. Polyploidy, a type of mutation that enhances the chromosome numbers is a widespread phenomenon in the evolution, development, agriculture, and diversity of flowering plants [36]. The main mechanism of colchicine is binding with alpha- and beta-tubulin dimers, which inhibits microtubule polymerization during the cell cycle (mitosis) in plant cells, after which chromosomes/chromatids migration is halted during the anaphase stage. It is acknowledged that cell division is blocked by colchicine mutagen, but its accurate mechanism in chromosomes and polyploidy induction of plants is still uncertain [37, 38]. Chemical mutagenic agents such as colchicine generate genetic variations, such as chromosome duplication (polyploidy) [39]. Polyploidy can generate variants with desired characteristics such as increased flower size, flowers with more showy colors and shapes, increased postharvest life, and greater resistance to abiotic or biotic stress [40]. It was reported that colchicine-treated plants exhibit drastic reduction in phenylpropanoid, phenylalanine, and phytohormone formation and vital increase in the genes expression which engaged in the induction of apoptosis and carcinogenesis agents which clarify why high mortality rate is associated with chromosome doubling when colchicine is used [38]. Genetic molecular markers, such as RAPD (randomly amplified polymorphic DNA), SSR (simple sequence repeats) and SRAP (sequence-related amplified polymorphism), offer considerable potential for rapid, efficient, and cost-efficient assessment of origin of regenerated plants and verifying their homozygosity status [41]. There are several studies on molecular markers detection of *P. graveolens*, mostly utilizing RAPD to examine the genetic diversity across various cultivated vanities [42]. Furthermore, Shasany et al. [43] used RAPD-marker to detect genetic variation among three cultivars; Algerian, Kelkar, as well as Bourbon. In vitro culture and molecular procedures have resulted in the creation of a new and wide paradigm in the using of mutation breeding for crop improvement, crop productivity, enhanced the characteristics, and germplasm innovation [44]. The recent research aimed to evaluate the impacts of γ-irradiation, laser and chemicals’ mutagen (colchicine & dinitroaniline) on the micropropagation of *Pelargonium graveolens*, as well as evaluate their effect on its oil chemical composition and the related genetic markers to conduct further selection.

## Results and discussion

Induced mutation or mutagenesis is defined as sudden heritable changes in the genome of an organism that do not result from genetic recombination but are induced by physical, chemical, or biological agents [45].

### Growth parameters

Results in Table 1 display the growth traits counting survival percentage, plant length (cm), branch numbers/plant, leaves number/plant, fresh weight/plant (g) and essential oil percentage in fresh leaves of the geranium mother plant and their induced different treatments. The data indicated that, the treatment with irradiation dose 40 Gy recorded the highest value of survival (66.67%) compared with the mother plants (16.67%). It was clearly observed in Table 1 for the plant height feature all over the induced plants that the dose level of 40 Gy recorded the greatest rate (30.50 cm), followed by the treatment of 5 mg/l dinitroaniline (24.83 cm), then the dose level of 20 Gy (24.50 cm), while 10 mg/l colchicine treatment had the lowest increase (16.00 cm) compared with the mother plants (10.00 cm). Concerning the branch number, it was obvious from the data in Table 1 that the highest branch number (7.00) was obtained in 10 mg/l colchicines followed by 10 mg/l dinitroaniline (6.00) treatments, while the lowest increase in branch numbers (1.33) was recorded in the treatments of 5.0 Gy, He-Ne laser for 15 and 30 min. Regarding the number of leaves, it can be concluded that the greatest leaves number was obtained by He-Ne laser for 5 min (17.67), while the lowest leaves number was recorded by 5 and 10 mg/l dinitroaniline (6.67). For the leaves’ fresh weight (g)/plant, it showed the same tendency, the maximum fresh weight (g/plant) was reported 14.36 g/plant in the gamma rays dose level of 40 Gy compared with the mother plants (1.26 g/plant).

**Table 1:** Impact of γ-irradiation, laser, colchicines, and dinitroaniline on survival percentage, plant length (cm), branch numbers/plant, leaves number/plant, fresh weigh/plant (g) and volatile oil percentage of geranium (*Pelargonium graveolens*) and its induced treatments in vitro

Different treatment		Survival %	Plant length (cm)	No. of branch/plant	Leaf No./plant	fresh weight/plant (g)	Essential oil %
Mother plants		16.67e ± 0.70	10.00e ± 0.70	1.00f ± 0.0	7.00h ± 0.30	1.501i ± 0.10	0.10e ± 0.01
Gamma rays (Gy)	5	33.33d ± 0.80	18.67c ± 0.30	1.33ef ± 0.15	9.67ef ± 0.70	6.25e ± 0.20	0.20d ± 0.02
	10	50.00c ± 0.60	19.70c ± 0.40	1.67ef ± 0.14	9.50fg ± 1.40	8.89d ± 0/3	0.34c ± 0.02
	20	60.00b ± 0.40	24.50d ± 0.50	2.00e ± 0.34	13.00bc ± 0.60	11.26c ± 0.20	0.35c ± 0.03
	40	66.67a ± 0.40	30.50a ± 0.30	4.00d ± 0.30	13.33bc ± 0.9	14.36a ± 0.80	0.40c ± 0.60
He-Ne Laser (min)	5	33.33d ± 0.80	20.50c ± 0.30	1.67ef ± 0.18	17.67a ± 1.20	12.62b ± 1.40	0.34c ± 0.03
	10	50.00c ± 0.60	19.33c ± 0.40	1.00f ± 0.09	12.00bcd ± 0.60	3.10h ± 0.10	0.38c ± 0.20
	15	50.00c ± 0.70	18.67c ± 0.60	1.33ef ± 0.12	11.67cd ± 0.30	5.67e ± 0.14	0.34c ± 0.20
	30	50.00c ± 0.50	19.67d ± 0.50	1.33ef ± 0.12	11.00de ± 0.50	4.21g ± 0.12	0.35c ± 0.02
Colchicine (mg/l)	5	50.00c ± 0.50	19.67b ± 0.40	5.00c ± 0.50	7.33gh ± 0.80	6.37e ± 0.52	0.50b ± 0.06
	10	50.00c ± 1.00	16.00c ± 0.6	7.00a ± 0.80	13.50b ± 0.90	12.69b ± 1.60	0.60a ± 0.03
Dinitro aniline (mg/l)	5	33.33d ± 0.60	24.83b ± 0.50	4.00d ± 0.24	6.67h ± 0.18	4.95f ± 0.70	0.13de ± 0.01
	10	50.00c ± 0.70	20.17c ± 0.90	6.00b ± 0.26	6.67h ± 1.80	5.81e ± 0.20	0.40c ± 0.03
L.S.D 0.05		5.01	3.01	0.82	2.62	0.74	0.016

Data are means (*n* = 3), standard errors (SE). Distinct different letters in the same column at *p* < 0.05 show a significant difference.

The current findings have the same opinion of that found by Aly et al. [46] and Gupta et al. [47] who accomplished that low doses of γ-radiation can improve the survival percentage of blackberry explants and strawberry under in vitro conditions, respectively. Furthermore, Billore et al. [48] displayed that significant positive impact on survival and growth of γ-irradiated shoots of *dendrobium* plants. Previous findings achieved from the other studies indicated that plantlets revealed a stimulatory impact of low doses gamma irradiation [19, 49]. There was a considerable impact of γ-irradiation low doses on the growth and yield characteristics of groundnut, while higher doses lead to a decrease in growth and yield [50]. Based on dose level, duration of exposure, and plant species, the effect of γ-irradiation on plants could be considered as a voluble tool in plant improvement and breeding programmes [51]. It is imperative to note that there is still much to learn about the mechanism of underlying how plants perceive laser as biostimulation. The observed morphological alteration in the cells and organisms can be primarily explained by the synergistic impact of several simultaneous bio-physico-chemical responses by the cotyledon and endosperm cell inside the seed upon absorption of laser light [26]. For laser rays the outcomes are in line with Perveen et al. [52] who found that applying laser rays at low-intensity to the seeds, seedling and plants causes stimulation. Furthermore, the absorption of laser rays by plants’ macromolecules initiates photosynthetic activity, which led to improvement in growth, biomass, and fresh weight [53]. In the same concern, Abou-Dahab et al. [54] established that the greatest leaves number and branch numbers of *Eustoma grandiflorum* plant were generated from the application of laser treatments for twenty minutes. Furthermore, Hassan et al. [55] illustrated that the irradiation power (20 mW and 50 mW) for five minutes significantly improved the vegetative growth parameters (sprouting days, plant height, leaves number, fresh and dry weight (g/plant), plant diameters, and leaf area), when compared with the mother plants of *Gladiolus grandiforus*. Also, Khamis et al. [56] reported that the germination rate of *Adansoina digitata* was significantly increased by low-power laser treatment (10 mW/two minutes). As well as, the root and leaves length were improved compared with the untreated and other laser treatments at different powers and time intervals. Previous study reported similar result; laser radiation improved plant organs such as leaves number and leave area, branches number, and umbels of fennel and coriander plants [57]. Furthermore, the metabolic processes in leaves linked to DNA replications, DNA repairs, cytokinesis, ribosome synthesis and translation might be impacted by the enhancement of phytochrome [58]. The mechanism of action for laser as an external physical factor, laser irradiation can promote the growth and development of seeds when the dose is appropriate. The mechanism may be that He-Ne laser irradiation changes the cell membrane function of seeds and even damages or destroys the structure, leading to increase permeability, which leads to enhance electrolyte extravasations and damage the seed structure. However, low doses of laser can also repair the membrane system of seeds which can effectively promote seeds germination [59].

Various chemical mutagen agents have been applied individually or compared by physical mutagen to generate wide spectra of mutant plant crops [60–62]. Chemical mutagenesis has been used to improve plant genetic diversity; this may be due to the fact that colchicine alters the polyploid level of one, two or three layers in the histogenesis layer, resulting in asynchrony of cell division and chromosome doubling events in these tissues [63]. In this study, colchicine and dinitroaniline increased survival percentage and other related potential agronomic traits. Colchicine mainly affects microtubules constancy through interacting with microtubules protein, reducing microtubule polymerisation and prevents the forming of a cytoskeleton. Due to the failure of microtubule production through mid-mitosis, chromosomes’ progress near the pole is impeded, leading to the formation of polyploid cells [39]. These improvements may be due to changes in gene expression, involving both the up- and down-regulation of procedures related to transport, biosynthesis, reception of primary and secondary metabolites, and different enzymes [64, 65]. Plant breeding is a novel strategy for producing unique mutations in crops either with a narrow genetic base or where the variability has already been exhausted [66]. Reduced mitotic activity in meristematic tissues, probable delay in mitosis, reduced auxin synthesis or enzyme activity, loss of plant assimilatory functions and chromosomal aberrations could be the cause of the marked mutagen impact on the root and shoot development of seedlings [67].

### Essential oil percentage

Essential oil results provided that the volatile oil percentage of fresh leaves ranged from (0.10 to 0.60%). The maximum essential oil yield in fresh leaves (ml/plant) was obtained in colchicine 10 mg/l (0.60 ml/plant), while the minimum increase was observed with dinitroaniline 5 mg/l (0.13%) treatments compared with the mother plant (0.10%) as indicated in Table 1. These data are sustained by previous studies that reported an enhancement of oil production with gamma irradiation in several plants, such as on *Raphanus sativus*-Red [18] and *Lavandula multifida* plants [68]. The recent results are in a harmony with Abou-Dahab et al. [54], who showed that the morphological traits were significantly improved when irradiation with red He–Ne irradiation output-power of 50 mW compared to the control.

### Essential oil composition

The changes caused by the induction of tetraploidy relies on the assumption that a lower ratio of nuclear membrane to chromatin resulted in more chromatin making contact with the nuclear membrane, thereby enhancing gene activity, improving water relations, hormone status, and photosynthetic rates. These factors may also have a positive impact on secondary metabolism and, consequently, on the production of phyto-pharmaceuticals [63]. In the same concern, Kumari and Singh [69] found that chemical mutagens affected the volatile oils content of *Ocimum basilicum* and increased by increasing their concentration. The findings of the GC-MS analyses of the geranium essential oil and its generated treatments from the mother plant; 40 Gy, 5, and 10 mg/l colchicine, and 10 mg/l dinitroaniline are provided in Table 2. The total recognized compounds are ranged from 79.35% in mother plant to 96.97% in 40 Gy treatment.

The main compounds (21 oxygenated monoterpenes) ranged from 71.67% in mother plants to 77.90% in the planted obtained from the different treatments, whereas the monoterpene hydrocarbons are presented (4 compounds) ranged from 0.67% in colchicine 10 mg/l to 2.63% in He-Ne treatment for 30 min. The sesquiterpenehydrocarbons (7 compounds) ranged from 1.99% in mother plants to 11.67% in 10 mg/l colchicine treatment and oxygenated sesquiterpene (9 compounds) ranged from 1.99% in mother plants to 11.15% in plants irradiated with 40 Gy. In addition, some another compounds like 5-aminopyrimidine-4, 6-diol which is considered a hydroxyl pyrimidine compound recorded 1.34% in mother plant and another two compounds 5-hepten-2-one,6methyl- which is considered a ketone (0.05%) and 6-methyl-hept-5-en-2-ol which is considered an alcohol (0.31%), they both obtained in the treatment 10 mg/l dinitroaniline. The geranium and its induced plants from the treatments; 40 Gy, 5, and 10 mg/l colchicine, and 10 mg/l dinitroaniline were varied in these contents of monoterpenes and sesquiterpenes, geranium is differed by its high content of oxygenated monoterpenes, which ranged from 71.67% in mother plants to 77.90% in 10 mg/l colchicine treatment. The content of volatile compounds stated as ratio was as follow: for 40 Gy (96.97%), 10 mg/l colchicine (95.73%), 10 mg/l dinitroaniline (94.38%), 5 mg/l colchicine (90.41%) and mother plant (79.35%). Table 2 presented the chemical compounds of the volatile oil. Here, 44 essential oil compounds and included 5 important identified compounds; citronellol, geraniol, citronellyl formate, l-linalool, and l-menthone were most predominant in all samples. Oil constituents of geranium and its induced plants from (40 Gy, 5, and 10 mg/l colchicine, and 10 mg/l dinitroaniline) as shown in Table 2 which indicated that the 40 Gy treatment recorded the highest percentage from citronellol, geraniol, citronellyl formate, L-linalool and l-menthone (26.18, 18.18, 6.66, 8.02 and 7.47%), respectively, while 10 mg/l colchicine treatment had different percentages (26.23, 17.23, 8.50, 7.75, and 7.08%), respectively. Furthermore, its percentages in 10 mg/l dinitroaniline treatment were (25.50, 18.10, 8.09, 7.06 and 6.91%) respectively, while they were (24.79, 19.20, 7.90, 7.89 and 7.28%), respectively and (25.04, 18.11, 7.82, 7.70 and 6.83%), respectively in 10 mg/l colchicine, and mother plants, respectively. In addition, dinitroaniline stimulated a lot of the essential oil compounds in *P. graveolens*, as 10 mg/l colchicine had 37 compounds recorded 94.38% while, the mother plants had 32 compounds recorded 79.35%. The ratio of citronellol to geraniol (C:G) in pelargonium species oil has been documented before [70]. In the recent investigation, the ratio of citronellol to geraniol (C:G) was achieved at all the tested samples. The ratio of citronellol to geraniol (C:G) is the main criteria to assess the perfumery value of geranium oil. The best quality of commercial geranium oil is characterized by an equal content of citronellol and geraniol (C:G = 1) [71]. Generally, laser light treatments exhibited a more pronounced effect on the essential oil content in *P. graveolens*. The current findings indicated that low γ-rays process influences the quantitatively and qualitatively the chemical composition of geranium. Also, the recent findings are in agreement with Kirkin et al. [72] who proved that volatile compounds in various essential oils are under specific environments and condition of each volatile compound submitted to various techniques of isomerization, oxidation, and hydroxylation, whilst submitted to γ-rays provide novel components. Gamma irradiation’s effects on the constituents of volatile oil can be influenced by a number of variables, including temperatures, sample conditions, dose level, dose rate and plants specie [18, 73]. In certain tetraploid plants grown in vitro by using colchicine; a positive correlation has been shown between the level of ploidy and the content of plant metabolites. Recent improvements have seen in several aromatic plants, such as *Melissa officinalis* L [74]. and *Salvia multicaulis* [75], enhance their medicinal ingredient content via chromosomes doubling.

**Table 2:** Impact of γ-irradiation, He-Ne laser, colchicine and dinitroaniline on essential oil composition percentage of geranium and its induced treatment in vitro

Peak No.	Oil components	RT (min)	Mother plants	20 Gy	Laser (30 min)	Colchicine (5 mg/l)	Dinitroaniline (10 mg/l)
1	3-methyl-1-propyl-	8.09	ND	ND	ND	ND	0.05
2	5-aminopyrimidine-4,6-diol	9.73	1.34	ND	ND	ND	ND
3	α -pinene, (-) -	10.31	0.54	0.89	0.75	ND	0.26
4	2,2-dimethyl-6-methyl-6vinyltetrahydropyran	11.25	ND	ND	ND	ND	0.04
5	5-hepten-2-one,6methyl-	11.62	ND	ND	ND	ND	0.05
6	6-methyl-hept-5-en-2-ol	12.98	ND	ND	ND	ND	0.31
7	β-pinene	13.13	1.82	1.91	1.88	0.67	0.98
8	à-Terpinolene	13.14	ND	ND	ND	-	0.38
9	1,8-Cineole	13.91	ND	ND	ND	0.15	ND
10	L-linalool	14.44	7.70	8.02	7.89	7.75	7.06
11	Rose oxide	14.62	0.73	ND	ND	1.63	ND
12	Cis-rose oxide	15.20	0.25	ND	ND	0.06	ND
13	Citronellal	15.22	ND	ND	ND	ND	0.04
14	p-menthan-3-one	15.42	ND	ND	ND	ND	0.08
15	l-menthone	15.72	6.83	7.47	7.28	7.08	6.91
16	Menthol, trans-1,3,cis-1,4-	15.73	0.25	0.44	0.50	ND	0.44
17	à terpineol	16.13	0.39	0.33	0.24	1.26	ND
18	Nerol	16.21	0.26	ND	ND	ND	1.15
19	Citronellol	16.25	25.04	26.18	24.79	26.23	25.50
20	à-citral	16.99	0.58	0.18	ND	ND	0.11
21	Geraniol	17.07	18.11	18.18	19.20	17.23	18.10
22	Citronellylformate	17.27	7.82	6.66	7.90	8.50	8.09
23	Geranylformate	17.63	2.78	2.50	4.10	3.50	3.10
24	Citronellyl acetate	17.71	0.11	0.15	0.05	0.19	0.17
25	à-copaene	18.54	0.21	0.25	0.30	2.66	1.07
26	à bourbonene	19.24	0.49	0.54	0.71	1.17	1.72
27	Geranyl acetate	19.59	0.32	0.61	0.89	0.55	0.59
28	Caryophyllene	20.01	0.37	4.65	3.37	2.17	2.41
29	Citronellyl propionate	20.17	ND	0.50	0.36	0.15	0.07
30	Germacrene- D	20.26	0.53	0.47	0.64	1.10	1.74
31	4-epi-cubedol	20.33	ND	3.67	1.91	0.05	2.33
32	ë-cadinene	20.62	0.21	0.81	1.92	2.30	0.07
33	Cadina-1,3,5-triene	21.00	0.18	0.65	ND	0.10	ND
34	Citronellyl butyrate	21.23	0.22	0.85	0.76	1.99	1.08
35	Eudesma-3,11-diene	21.66	ND	0.22	0.36	2.17	1.05
36	Geranyl butyrate	23.59	0.28	3.36	0.46	1.63	1.33
37	(-)-caryophyllene oxide	24.51	0.62	1.18	0.98	0.04	ND
38	Veridiflorol	25.70	ND	ND	ND	1.52	2.96
39	Rosifoliol		0.11	1.42	0.29	0.81	0.45
40	Cubenol		0.14	1.29	0.36	1.68	1.87
41	10-epi-ҫ-eudesmol		0.74	1.67	1.01	1.05	2.33
42	ҫ-eudesmol		0.17	1.92	0.76	0.03	0.35
43	Eudesm-4(14)-en-11-ol		0.07	ND	0.37	0.16	0.09
44	Geranyltiglate		0.14	ND	0.38	0.15	0.05
	To Total Identified		79.35	96.97	90.41	95.73	94.38
	Total Unidentified		20.65	3.03	9.59	4.27	5.62
	Monoterpene hydrocarbons		2.36	2.8	2.63	0.67	1.67
	Oxygenated monoterpenes		71.67	75.43	74.42	77.9	73.86
	Sesquiterpene hydrocarbons		1.99	7.59	7.3	11.67	8.06
	Oxygenated Sesquiterpene		1.99	11.15	6.06	5.49	10.43
	Other compounds		1.34	ND	ND	ND	0.36

*ND *Not Detected

### Molecular genetics identification

Various types of radiation have been used to attain the required genetic heterogeneity in a range of ornamental plants. The determination of dose for chemical mutagens is often made by varying the concentration and duration of treatment.

### Randomly amplified polymorphic DNA (RAPD) markers

**Fig. 1:**
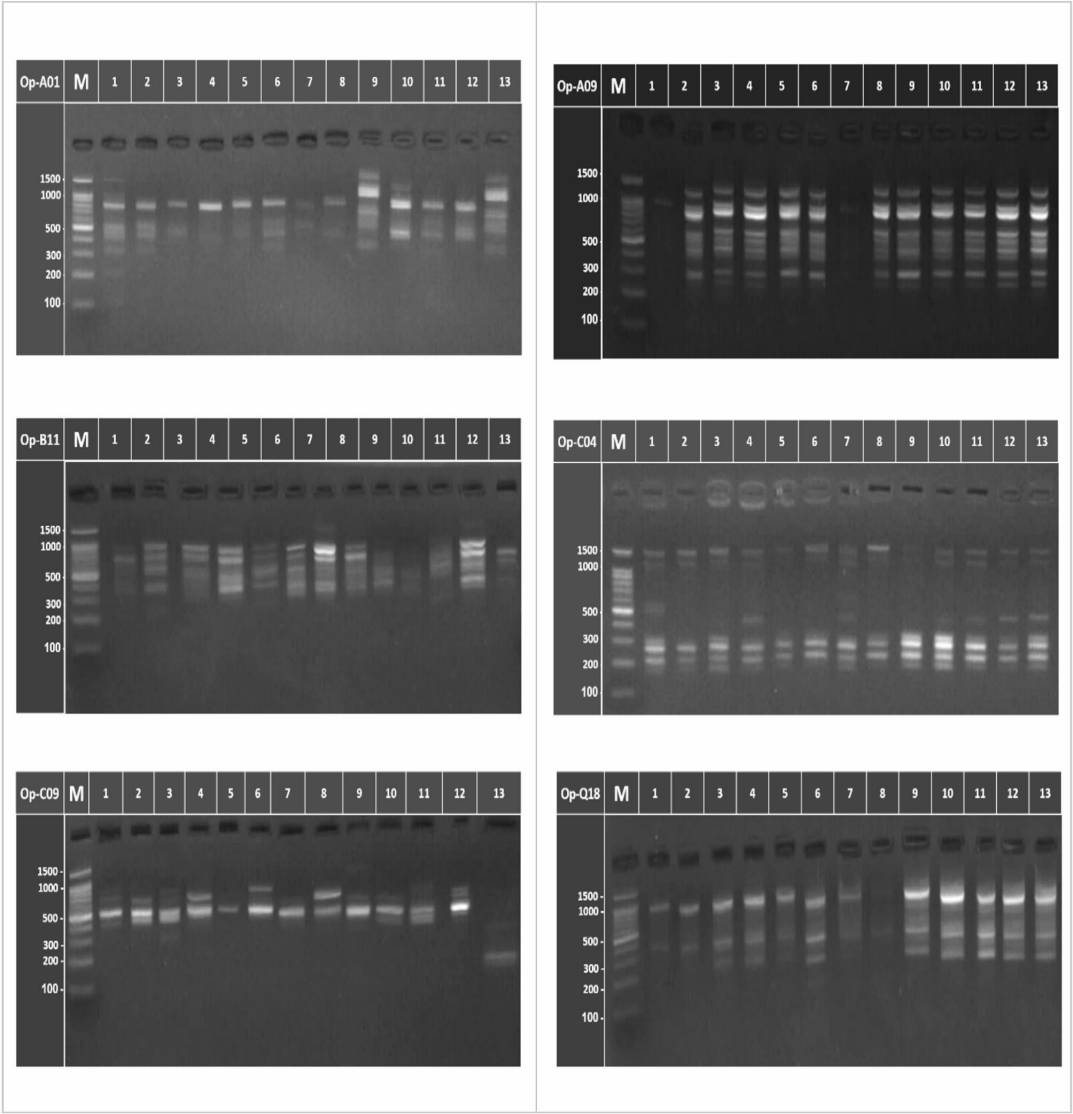
RAPD-PCR analysis of the mother plants of *P. graveolens* and its induced plants from different treatments in vitro (1 = Mother plant, 2 = 5 Gy, 3 = 10 G 4 = 20 Gy, 5 = 40 Gy, 6 = He-Ne laser 5 min, 7 = He-Ne laser 10 min, 8 = He-Ne laser 15 min, 9 = He-Ne laser 30 min, 10 = 5 mg/l colchicine, 11 = 10 mg/l colchicine, 12 = 5 mg/l dinitroaniline, 13 = 10 mg/l dinitroaniline) and M is a 100 bp ladder marker (1500, 1000, 900, 800, 700, 600, 500, 400, 300, 200, and 100 bp)

The RAPD procedure is quite simple to apply and doesn’t need labeled probes restriction digestion, hybridization or previous sequence knowledge and the utilizing of hazardous detection chemicals. The dominating nature and low repeatability of RAPD marker is drawbacks [76]. The analyses of RAPD markers presented qualitative and quantitative differences amongst the mother plant and induced plants from different treatments (Fig. 1; Table 3). Polymorphism levels varied from one primer to another, the OP-B11 primer displayed low level of polymorphisms (50.00%), however, OP- C04 (66.67%) and OP- Q18 (58.71%) primers revealed reasonable level of polymorphisms. On the other hand, OP-A09 (90.00%), OP-A01 (93.75) and OP-C09 (100.00) primers demonstrated higher levels of polymorphisms. The number of total amplified fragments (TAF), polymorphic fragments (PF), monomorphic fragments (MF) and specific markers (SM) for each treatment using six primers are revealed in Table (3). The OP-A01 primer generated sixteen fragments with molecular size ranging from 110 to 1401 bp (Fig. 1). Fifteen fragments were polymorphic (93.75%) and seven of them were treatment-specific markers at (884,199,110 bp) for mother plant, (456 bp) for 10 Gy (427 bp) for He-Ne laser 5 min, (706 bp) for He-Ne laser 30 min and (642 bp) for 10 mg/l dinitroaniline, whereas just one fragment was presented in all the treatments which is considered as common fragment. Ten DNA fragments were generated from OP-A09 primer with molecular size ranging from 216 to1213 bp, nine of them were polymorphic (90.00%) and one fragment was found in all treatments which are considered as familiar fragments. OP-B11 primer showed eight DNA fragments with molecular size from 360 to1570 bp, 4 fragments were polymorphic (50.00%), and another four fragments were found in all the treatments which are considered as common fragments. The OP-C04 primer presented in nine DNA fragments with molecular sizes ranging from 175 to 1457 bp, six fragments from them were polymorphic (66.67%) and one of them were treatments-specific marker at (510 bp) for the mother plant. Whereas, the other three fragments which are considered as common fragments in all the treatments. The OP-C09 primer produced 10 DNA fragments with molecular sizes from 184 to 990 bp.

Ten of these were polymorphic (100.00%) and six of them were treatments-specific markers at (314 bp) for 10 mg/l dinitroaniline, (746 bp) for He-Ne laser 10 min, (402 bp) for He-Ne laser 15 min and (429, 323, 184) for 10 mg/l NDA. The OP-Q18 primer resulted in seven DNA fragments at molecular size ranging from 212 to 1521 bp, six fragments were polymorphic (85.71%), while one fragment was presented in all the treatments which are considered as common fragments.

**Table 3:** Treatments-specific RAPD and ISSR markers for the mother plant and its induced treatments of *Pelargonium graveolens in vitro*

Primers code	Range of M.S.	TAF	MF	PF	SM	Polymorphism (%)
RAPD primers						
OP-A01	110–1401	16	1	15	7(884,199,110)-(0)-(456)-(0)-(0)-(427)-(0)-(0)-(706)-(0)-(0)-(0)(642)bp	93.75
OP-A09	216–1213	10	1	9	0	90.00
OP-B11	360–1570	8	4	4	0	50.00
OP-C04	175–1457	9	3	6	1 (510)-(0)-(0)-(0)-(0)-(0)-(0)-(0)-(0)- (0)-(0)-(0)-(0) bp	66.67
OP-C09	184–990	10	0	10	6 (0)-(0)-(314)-(0)-(0)-(0)-(746)-(0)-(402)-(0)-(0)-(0)-(429,323,184) bp	100.00
OP-Q18	212–1521	7	1	6	0	85.71
Total RAPD primers		60	10	50	14	
ISSR primers						
14 A	256–875	10	2	8	4(0)-(0)-(409)-(0)-(0)-(875)-(440)-(0)-(0)-(0)-(805)-(0)-(0)bp	80.00
44B	287–1203	16	1	15	7 (0)-(393,306)-(298)-(1203)-(1009)-(0)-(0)-(0)-(0)-(0)-(0)-(1184,583)-(0)bp	93.75
HB-09	348–1551	10	5	5	3 (0)-(590)-(0)-(0)-(0)-(0)-(0)-(364)-(358)-(0)-(0)-(0)-(0) bp	50.00
HB-11	207–1591	19	1	18	10 (408,357)-(1176,267)-(1007)-(0)-(0)-(0)-(1591)-(0)-(1517)-(1097)-(207)—(0)-(1079) bp	94.74
HB-12	192–1199	10	4	6	2 (0)-(0)-(0)-(0)-(0)-(873)-(0)-(0)-(0)-(0)-(0)-(192)-(0) bp	60.00
HB-15	322–2940	8	1	7	1 (0)-(0)-(0)-(0)-(0)-(0)-(0)-(0)-(2940)-(0)-(0)-(0)-(0)bp	87.50
Total ISSR primers		73	14	59	27	
Total		133	24	109	41	

*TAF *Total Amplified Fragments, *MF *Monomporphic Fragments, *PF* Polymorphic Fragments, *SM* Specific Markers

### Genetic similarity and cluster analysis based on RAPD markers

The RAPD data were used to estimate the genetic similarity values among the mother plant and its induced mutants of geranium by using UPGMA computer analysis (Table 4; Fig. 1). According to the RAPD results, the most closely related treatments to the mother plant genotype were 40 Gy and He-Ne laser 15 min (Table 4), which had the maximum similarity index (1.00) and (0.979) respectively. However, the most distant genotype treatment from the mother plant genotype was 10 Gy, with a lower similarity index (0.539), while the two treatments found extremely far were 5 mg/l colchicine and He-Ne laser 30 min and also 10 and 5 mg/l colchicine (similarity index of 0.035). On the other hand, there was no similarity between 10 mg/l colchicine and 5 mg/l dinitroaniline treatment (Table 4). In the current investigation, there was a noteworthy variation between the treated plants and the mother plant. Several fragments were missing in the different treatments, and other new fragments appeared. For example, the primer OP-A01, fragments (884,199, and 110 bp) were missing from the treated plants, whereas novel bands (456, 427, 706 and 642 bp), appeared in (10 Gy, 5 mg/l colchicine, He-Ne laser 30 min and 10 mg/l dinitroaniline treatments), respectively. A similar variation was also shown for primer OP-C04, fragment (510 bp) was missing in all treated plants, while a new band, (431 bp), appeared in (20 Gy, He-Ne 10 min, 5 mg/l colchicine, 10 mg/l colchicine, 5 mg/l dinitroaniline and 10 mg/l dinitroaniline treatments (Table 3). The dendrogram for the genetic relationship between the different treatments and the mother plant is exhibited in Figs. 1 and 3A, depending on the RAPD and ISSR markers which separated them into two main clusters. The first cluster included mother plant, whilst the second cluster consisted of all the plants induced from the different treatments.

**Table 4:** Similarity value (pair wise comparison) among the mother plant and its induced plants from different treatments of *P. graveolens in vitro* based on RAPD markers

Treatments		Proximity Matrix											
		Mother plants	Gamma rays (Gy)				He-Ne Laser (min)				Colchicine (mg/l)		Dinitroaniline (mg/l)
			5	10	20	40	5	10	10	30	5	10	5
Mother plants													
Gamma rays (Gy)	5	0.539											
	10	0.638	0.094										
	20	0.922	0.263	0.236									
	40	1.000	0.294	0.263	0.133								
He-Ne Laser (min)	5	0.782	0.236	0.144	0.377	0.263							
	10	0.737	0.850	0.778	0.676	0.746	0.697						
	15	0.979	0.262	0.232	0.262	0.119	0.155	0.717					
	30	0.613	0.223	0.330	0.429	0.395	0.330	0.824	0.367				
Colchicine (mg/l)	5	0.661	0.194	0.173	0.264	0.293	0.239	0.643	0.338	0.035			
	10	0.753	0.223	0.135	0.292	0.249	0.135	0.590	0.367	0.253	0.035		
Dinitroaniline (mg/l)	5	0.753	0.154	0.135	0.154	0.322	0.200	0.668	0.293	0.253	0.099	0.000	
	10	0.812	0.436	0.403	0.502	0.473	0.340	0.812	0.519	0.266	0.180	0.266	0.327

### Inter simple sequence repeats (ISSRs) markers

**Fig. 2:**
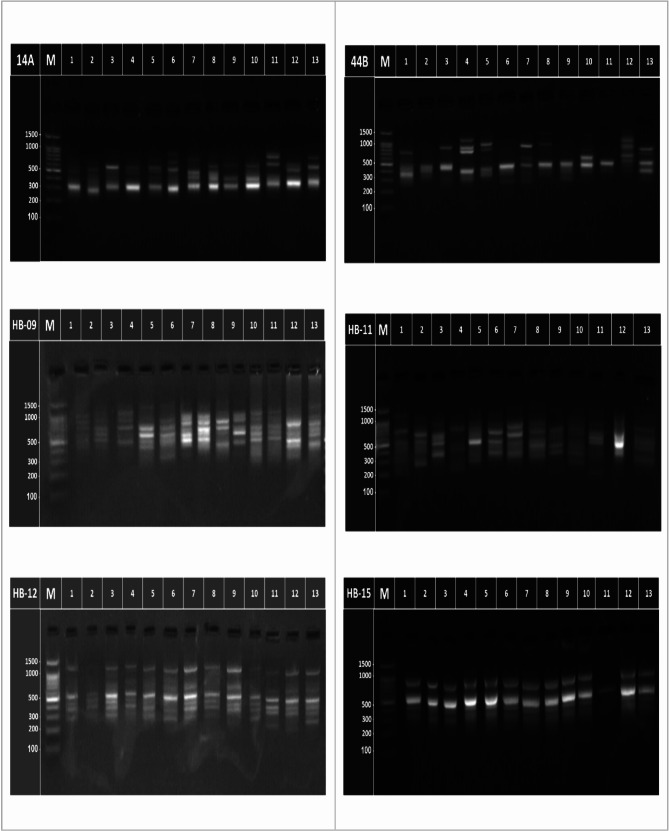
ISSR-PCR analysis of the mother plants of *P. graveolens* and its induced plantsfrom different treatments (1 = Mother plant, 2 = 5 Gy, 3 = 10 G 4 = 20 Gy, 5 = 40 Gy, 6- He-Ne laser 5 min, 7 = He-Ne laser 10 min, 8 = He-Ne laser 15 min, 9 = He-Ne laser 30 min, 10 = 5 mg/l colchicine, 11 = 10 mg/l colchicine, 12 = 5 mg/l dinitroaniline, 13 = 10 mg/l dinitroaniline), and M is a 100 bp ladder marker (1500, 1000, 900, 800, 700, 600, 500, 400, 300, 200, and 100 bp)

The six ISSR primers succeeded in amplifying DNA fragments for the mother plant and its induced plants from different treatments of *P. graveolens* (Fig. 2; Table 3). Polymorphic levels varied from each one primer to another, i.e., 44B and HB-11 primers exhibited high levels of polymorphism (93.75 and 94.74%), respectively, while (14 A, HB-12, and HB-15) primers induced moderate levels of polymorphism (80.00, 60.00 and 87.50%), respectively. In addition, the HB-09 primer generated a lower level of polymorphism (50.00%) as demonstrated in (Table 3).

The 14 A primer illustrated four affirmative specific-markers, one of them 409 bp for 10 Gy treatment, a 875 bp fragment for He-Ne laser 5 min treatment, another 440 bp fragment for He-Ne laser 10 min treatment, and one more specific-marker, 805 bp, for 10 mg/l colchicine. Additionally, two more bands, 500 and 303 bp, were found in all samples. The 44B primer produced 7 positive specific markers, 2 of them 393 and 306 bp for 5 Gy treatment, one marker, 298 bp, for 20 Gy, a 1203 bp fragment for 40 Gy, a 1009 bp fragment for He-Ne laser 5 min, two markers, 1184 and 583 bp for 5 mg/l dinitroaniline. One common fragment, 497 bp, was generated in all examined treatments. The HB-09 primer induced three affirmative specific-markers at 590 bp for 5 mg/l dinitroaniline, one marker at 364 bp for the He-Ne laser 15 min treatment and one specific-marker at 358 bp for the He-Ne laser 30 min, whereas the five fragments were found in all samples. On the other hand, the HB-11 primer generated ten positive specific-markers; two of them (408 and 357 bp) for the mother plant, two markers (1176 and 267 bp) fragments for the 5 Gy treatment, a 1007 bp marker for 10 Gy treatment, one positive specific-marker (1591 bp) for the He-Ne laser 10 min, a 1517 bp for He-Ne 30 min sample, one specific-marker (1097 bp) for the 5 mg/l colchicine, another positive specific-markers (207 bp) for the 10 mg/l colchicine sample and the last specific-marker (1079 bp) for the 10 mg/l dinitroaniline sample, while one common fragment (502 bp) was found in all treatments. The HB-12 primer showed two positive specific-markers: a 873 bp fragment for H-Ne laser 5 min and a 192 bp fragment for 5 mg/l dinitroaniline, while four fragments, each 533, 450, 380 and 286 bp, were found in all samples. The HB-15 primer showed one affirmative specific- marker, a 2940 bp fragment for He-Ne 30 min sample, while one more band, 322 bp, was present in all treatments.

**Table 5:** Similarity value (pair wise comparison) among the mother plant and its induced plants from different treatment of *P. graveolens in vitro* based on ISSR markers

Treatments		Proximity Matrix											
		Mother plants	Gamma rays (Gy)				He-Ne Laser (min)				Colchicine		Dinitroaniline (mg/l)
			5	10	20	40	5	10	15	30	5	10	5
Mother plants													
Gamma rays (Gy)	5	0.611											
	10	0.694	0.522										
	20	0.061	0.909	0.476									
	40	0.140	0.453	0.531	0.163								
He-Ne Laser (min)	5	0.421	0.496	0.453	0.332	0.051							
	10	0.574	0.653	0.237	0.362	0.304	0.234						
	15	0.314	0.265	0.214	0.476	0.040	0.212	0.113					
	30	0.579	0.655	0.377	0.496	0.433	0.364	0.392	0.377				
Colchicine (mg/l)	5	0.821	0.651	0.607	0.738	0.286	0.332	0.487	0.345	0.377			
	10	0.821	0.780	0.738	1.000	0.408	0.332	0.611	0.345	0.496	0.345		
Dinitroaniline (mg/l)	5	0.845	0.809	0.538	0.772	0.474	0.622	0.657	0.421	00.337	0.421	0.538	
	10	0.769	0.845	0.694	0.694	0.187	0.337	0.584	0.464	0.272	0.118	0.118	0.000

### Genetic similarity and cluster analysis based on ISSR markers

The DNA markers can be generated with the ISSRs without prior information of the genomic sequences, and they have been shown to be a dependable, quick, easy, affordable, and adaptable set of markers. Plant genetic analysis frequently employs ISSR markers, which are also helpful in research of genetic diversity, phylogeny, gene tagging, genome mapping and evolutionary biology [77]. According to the ISSR data, the most closely related plants to the mother plant which were induced from the treatment of 5 Gy (Table 5), which had the maximum similarity index (0.845). Otherwise, the most distant sample from the mother plant was 20 Gy treatment, which had low similarity index (0.061), while the two other samples located very far were the 40 Gy and He-Ne laser 5 min (similarity index of 0.051). On the other hand, the most closely two samples were 20 Gy and 10 mg/l colchicine, with a high similarity index (1.00), while there was no similarity between 5 and 10 mg/l dinitroaniline samples (Table 5). A vital diversity among the treated sample and the mother plant, for primer HB-11, fragments (408 and 357 bp) were missing in the treated plants, whereas novel bands, (1176 and 267 bp), appeared in the 5 Gy sample, and (1007, 1591, 1517, 1097, 207, and 1079 bp) appeared in the 5 Gy, He-Ne laser 10 min, He-Ne laser 40 min, 5 and 10 mg/l colchicine and 10 mg/l dinitroaniline samples, respectively (Table 3).

**Fig. 3:**
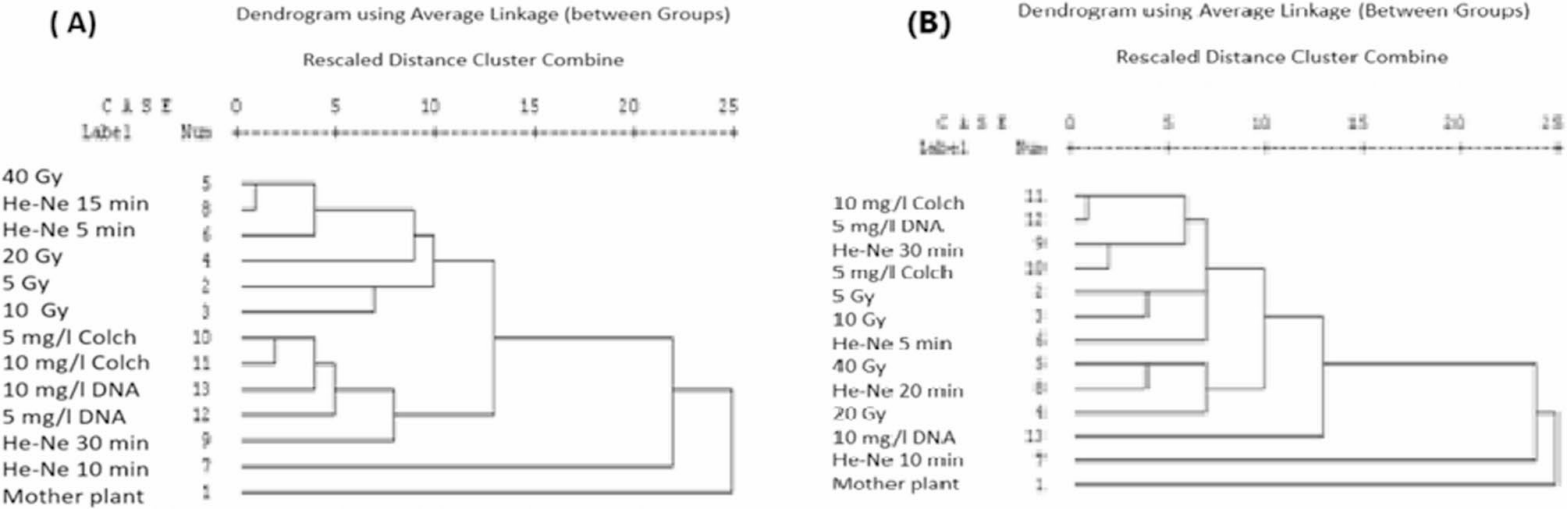
A dendrogram illustrates the genetic distance for the mother plant and its induced plants from different treatments in vitro of geranium (*Pelargonium graveolens*) based on RAPD (**A**) and ISSR (**B**) analysis

Since the treatments under investigation appear to be genetically distinct, the significance level of polymorphic suggests that the studied plants are genetically different, indicating that these markers technique were appropriate for examining the genetic diversity between different treatments compared to the mother plant. The effectiveness of ISSR and RAPD as genetic markers was applied to evaluate the genetic variation of *P. graveolens* different treatments. The dendrogram based on ISSR-PCR markers (Fig. 2 and 3B) separated the mother plant and its putative mutants of geranium treatments into two main clusters according to UPGMA analyses. The first cluster contained only the mother plant genotype, while the second cluster is divided into two major subclusters. The first subcluster included all colchicine and dinitroaniline treatments, while the other subgroup included all gamma radiation and laser treatments.

**Fig. 4:**
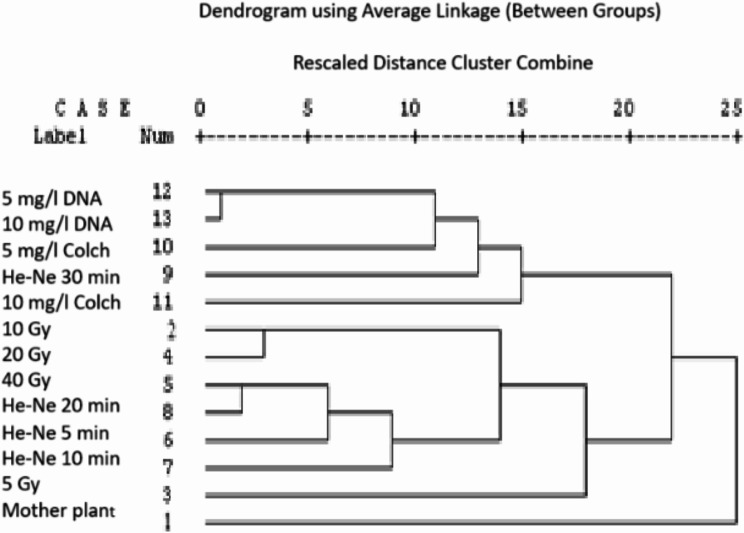
A dendrogram illustrates the genetic distance for the mother plant and its induced plants from different treatments in vitro of geranium (*Pelargonium graveolens*) genotypes based on over-combination of RAPD and ISSR analysis

### Genetic similarity and clustering analysis based on RAPD and ISSR markers

The mother plant and its plants induced from different treatments are distributed overall based on the RAPD and ISSR banding patterns, which are differ from one to another, that might be owing to each procedure amplifying various parts from of the genome. Therefore, it is better for the combination of the banding patterns of the two methods to utilize more segments of the genome that will improve the strength of the consensus tree. The collected data as indicated in Table 6 demonstrated that the most closely related treatments to the mother plant genotype was 10 mg/l dinitroaniline which had the maximum similarity index (1.00). Alternatively, the most distant treatment from the mother plant was the 5 Gy treatment, with a low similarity index (0.672), while the two samples found very far were 5 mg/l colchicine and both of (He-Ne laser 30 min and 10 mg/l colchicine), with similarity index of (0.082 and 0.051), respectively. On the other hand, there was no similarity between the treatments of 40 Gy and He-Ne laser 15 min (Table 6). The dendrogram based on RAPD and ISSR-PCR techniques (Fig. 4) divided the mother plant and its putative treatments of geranium genotype into two main clusters.

**Table 6:** Similarity value (pair wise comparison) among the mother plant *P. graveolens *ofand its induced plants via mutagen agents based on the combination of RAPD and ISSR markers

Treatments		Proximity Matrix											
		Mother plants	Gamma rays (Gy)				He-Ne Laser (min)				Colchicine		Dinitroaniline (mg/l)
			5	10	20	40	5	10	15	30	5	10	5
Mother plants													
Gamma rays (Gy)	5	0.672											
	10	0.806	0.214										
	20	0.717	0.581	0.320									
	40	0.785	0.377	0.387	0.077								
He-Ne Laser (min)	5	0.783	0.341	0.227	0.380	0.125							
	10	0.809	0.951	0.670	0.643	0.643	0.585						
	15	0.880	0.243	0.187	0.357	0.000	0.122	0.513					
	30	0.721	0.412	0.359	0.513	0.451	0.354	0.783	0.395				
Colchicine (mg/l)	5	0.892	0.371	0.316	0.477	0.280	0.251	0.693	0.351	0.082			
	10	0.978	0.460	0.341	0.632	0.306	0.153	0.717	0.377	0.345	0.051		
Dinitroaniline (mg/l)	5	0.987	0.435	0.262	0.412	0.412	0.376	0.805	0.355	0.271	0.167	0.132	
	10	1.000	0.702	0.587	0.682	0.382	0.349	0.886	0.575	0.247	0.087	0.168	0.160

 The first subcluster included all colchicine and dinitroaniline treatments, while the other subgroup included all gamma radiation and laser treatments. In this concern, previous authors have assumed that the absence of intraclonal RAPD polymorphisms can’t guarantee genetic stability due to significant differences, as genomic mutation could be missed [86]. In the current investigation, the obtained plants from different mutant agents and their mother plant line displayed intensive polymorphism, as revealed by this analysis (Table 3). Findings of morphological, RAPD and ISSR markers demonstrated that the plants induced from different mutagen agents were not always as close (Tables 4 and 5) at the molecular levels. The similarity index (Tables 4 and 5) illustrated low percentages of similarity between the plants induced by different mutagens agents and their mother plant. These divergent induced plants could be utilized in plant breeding programmes for gene pyramiding of yield and oil content attributes. Accordingly, ISSR primers are applied to confirm the genetic variations at the molecular level and to find several specific-markers associated to induced plants that might be used for genetic fingerprinting of those treatments. In current study, some samples proved unique distinct molecular and agronomical characteristics. The treatment 5 Gy showed the highest values for survival percentage and fresh weight, which could be fingerprinted with 2 positive specific-markers, 393 and 306 bp for primer 44B or 590 bp for HB-09 or 1176 and 267 for HB-11. On the other hand, 40 Gy showed the highest plant length value which might be associated to a specific unique band, 1009 bp produced by primer 44B. For the sample treated by He-Ne laser 5 min induced the greatest leaves number/plant which could be associated to a specific unique band 17.67 bp produced by primers 44B. Similarly, 5 mg/l colchicine distinguished itself for a high volatile oil percentage of 0.5% associated with the unique band of 1097 bp produced by primer HB-11, which could be used as a marker for this character and, also, as a genetic fingerprint for this treatment.

According to the treatment, 10 mg/l colchicine showed the highest branching and considered the only treatment which had 1,8-cineole as a volatile oil, which could be linked to two specific unique bands, 805 bp produced by primer 14 A and 207 bp for primer HB-11. Both, RAPD and ISSR fingerprinting for the other treatments proved sufficient number of unique bands corresponding to each mutant agent, impressive which would be very useful for mutant characterization and identification. When comparing ISSR to RAPDs, the overall number of polymorphic fragments is greater in ISSR than RAPD. Compared to the other random primers as RAPDs, the ISSRs actually have high ability to detect polymorphism and offer great potential for determining intra-and inter genomic diversity [78].

Number of polymorphisms discovered with each marker may be a better indicator of the ability to resolve genetic diversity. The amount of polymorphisms found with each marker may have direct behavior on the capacity to resolve genetic diversity among various genotypes. According to Hassan et al. [55], laser treatment in *Gladiolus grandiforus* corms impacted by laser and the gene expression caused color or abnormal features in leaf and flower. As well as several alleles which could result in abnormalities such as mutations in the allele with 410 bp generated by SSR.

The recent data are in accordance with Hiremath et al. [79], who utilized SSR for studying the genetic diversity and structure analysis for efficient utilization and sustainable management of gladiolus germplasm. Also, El Sherif et al. [33] investigated that the alteration in the ISSR profile induced by laser treatments in different jojoba lines could be interpreted as changes in the genomic DNA template with direct comparisons between these genetic outcomes and variations in other parameters. Additionally, Shehata et al. [80] found that the greatest quantity of GTS was 40% at a laser power of 20 mW for 120 min, while the least quantity was obtained at 22.5% at 5 mW power for thirty seconds and the mother plants of *Phaseolus vulgaris.* Gorbatova et al. [81] established that the genetic configuration of the varieties was an imperative feature in their reaction with the low doses of gamma rays. As well as Abd El-Aziz et al. [82] found that gamma rays were successful for inducing desirable changes in okra at the two phenotypic and molecular levels by RAPD marker. Azzam et al. [83] indicated that IRAP and SRAP markers successfully detected DNA polymorphism between the un-irradiated and γ-irradiated cowpea M2 and recommend utilizing gamma rays as a significant way to improve cowpea genotypes, particularly since linked by molecular markers. In the same regard, Raina et al. [84] provided that enhancing the genetic diversity with gamma rays was found to utilize biochemical, physiological, and molecular profiling. On the other hand, Dong et al. [85] successfully induced and obtained tetraploid plants of black currant by using colchicine to treat sterile seeds of black currant. This is helpful to reveal the molecular mechanism of polyploid formation and provide a molecular basis for genetic engineering breeding of black currant and the localization of key regulatory gene. In the same concern, Din et al. [86] provided precious insight into the genetic composition of the induced mutants and emphasized their prospective for improving chrysanthemum-breeding programmes through gamma rays and chemical agents.

### Principal component analysis (PCA) for RAPD

The PCA performed on the RAPD dataset provides a comprehensive understanding of the underlying relationships among the variables involved. This dataset comprises 60 observations across 13 variables, which are crucial for analyzing genetic diversity through Random Amplified Polymorphic DNA (RAPD) techniques. The PCA aims to reduce the dimensionality of the dataset while retaining as much variance as possible, thereby simplifying the complexity of the data without losing significant information.

**Fig. 5:**
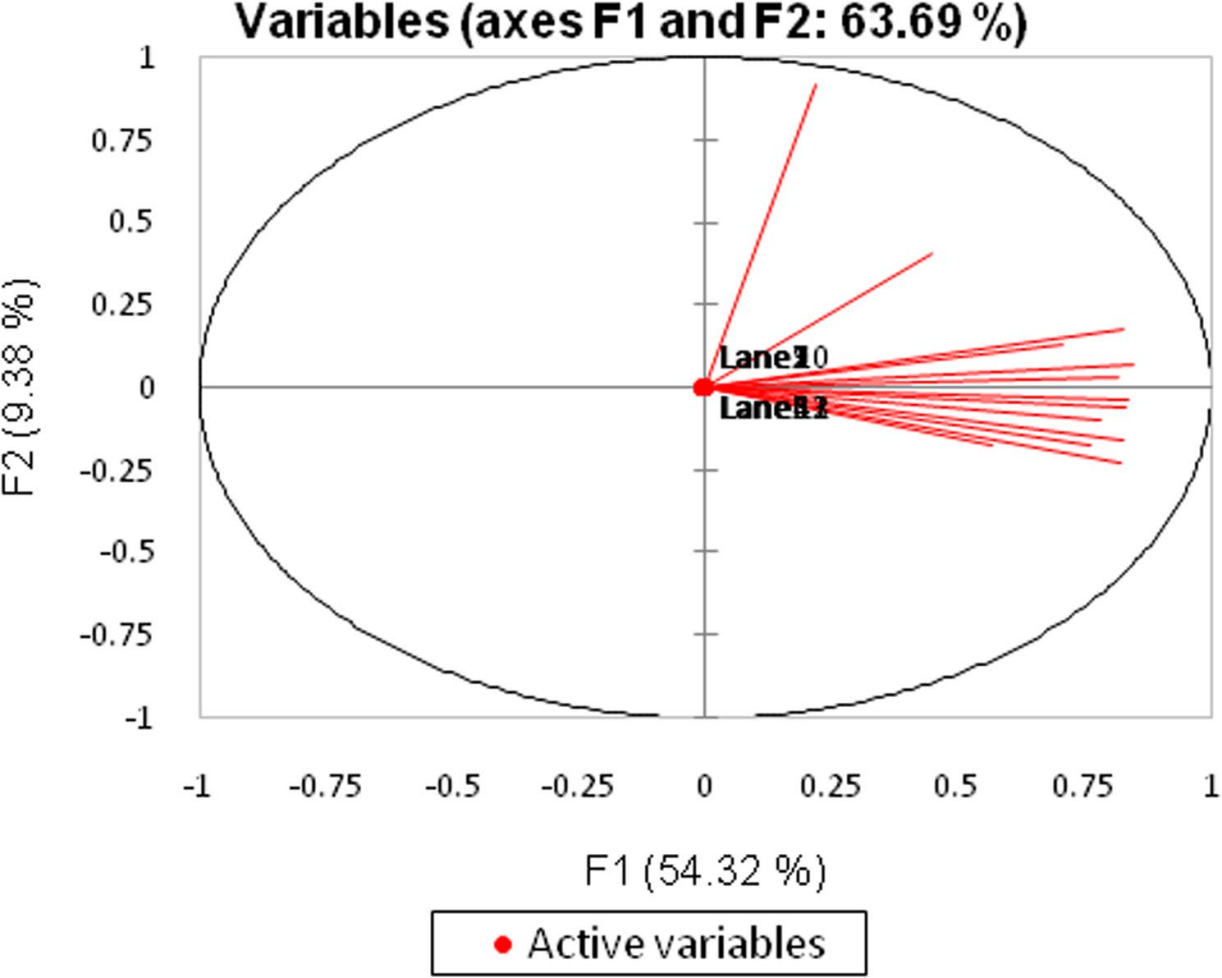
Principal component analysis (PCA) scatter diagram demonstrating the genetic diversity expressed by grouping *Pelargonium graveolens* different treatments established on the analysis RAPD marker polymorphism and by blotting the two principal components

In this analysis, the first principal component (F1) accounts for an impressive 54.32% of the total variance, indicating that a substantial portion of the data’s variability can be explained by this single component. The cumulative variance explained by the first two components reaches 63.69%, suggesting that these components capture the essential patterns within the data (Fig. 5). This reduction in dimensionality is particularly beneficial for subsequent analyses, such as clustering or classification, as it allows researchers to focus on the most informative aspects of the data.

The Kaiser-Meyer-Olkin (KMO) measure of sampling adequacy, which stands at 0.846, further supports the appropriateness of conducting PCA on this dataset. A KMO value above 0.6 is generally considered acceptable, indicating that the sample size is sufficient for the analysis. Additionally, Bartlett’s test of sphericity yields a chi-square value of 538.821 with a *P*-value less than 0.0001, confirming that the correlation matrix is significantly different from an identity matrix. This result reinforces the validity of the PCA, as it indicates that there are meaningful correlations among the variables. The analysis also provides insights into the contributions of each variable to the principal components. By examining the factor loadings, researchers can identify which variables are most influential in shaping the principal components. This information is crucial for understanding the data structure and can guide further investigations into the genetic diversity represented in the RAPD analysis.

### Principal component analysis (PCA) for ISSR

The PCA conducted on the ISSR dataset offers a comprehensive examination of the relationships among the variables that are critical for understanding genetic diversity. This dataset consists of 73 observations and 13 variables, which are essential for analyzing genetic variation through ISSR techniques. The primary objective of the PCA is to reduce the dimensionality of the dataset while preserving as much variance as possible, thereby simplifying the complexity of the data without sacrificing significant information.

**Fig. 6:**
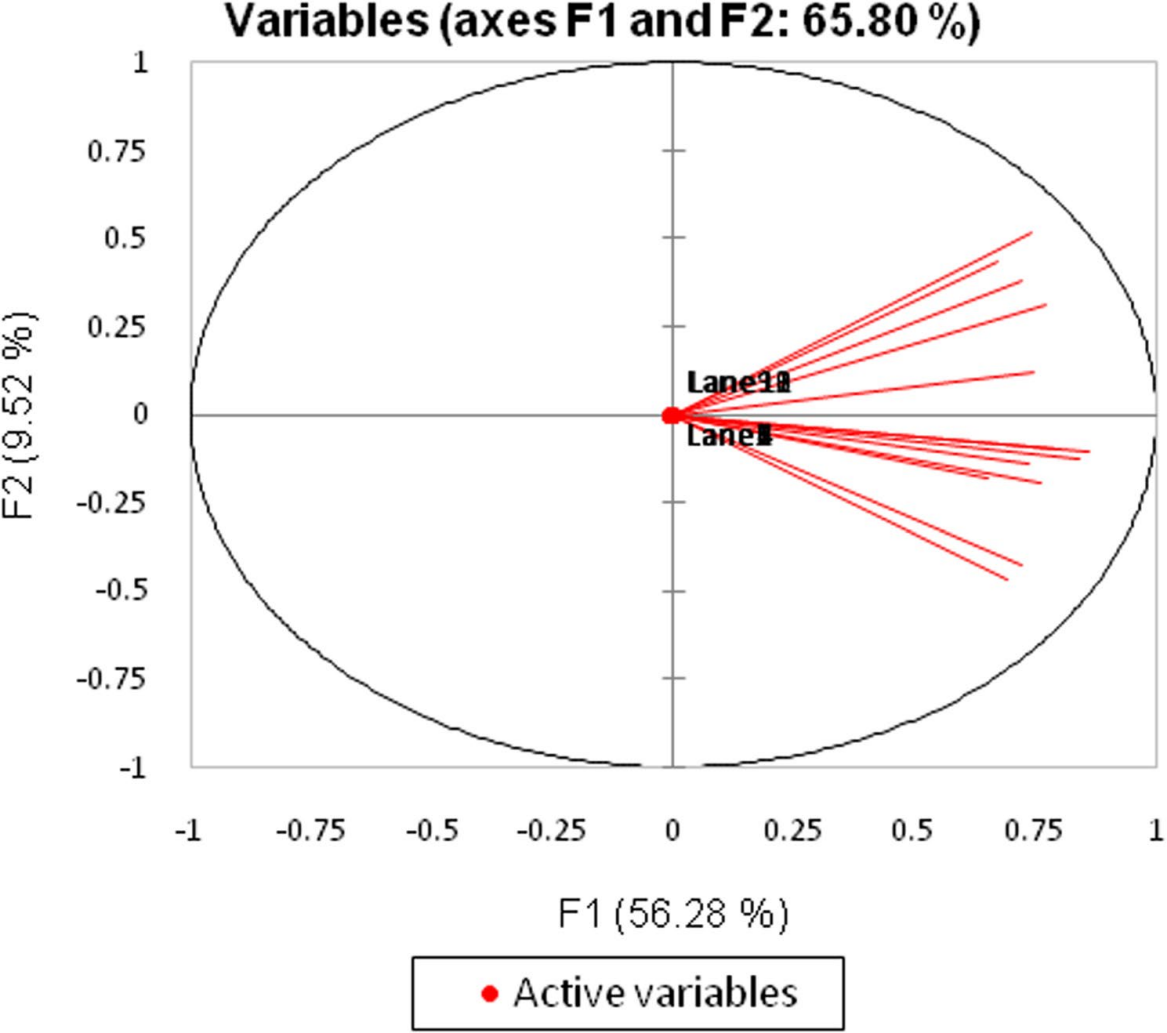
Principal component analysis (PCA) scatter diagram demonstrating the genetic diversity expressed by grouping *Pelargonium graveolens* at different treatment established on the analysis of ISSR marker polymorphism and by blotting the two principal components

In this analysis, the first principal component (F1) accounts for a substantial 56.28% of the total variance, indicating that a significant portion of the variability in the data can be explained by this single component (Fig. 6). The cumulative variance explained by the first four components reaches 77.36%, suggesting that these components capture the essential patterns within the data. This reduction in dimensionality is particularly advantageous for subsequent analyses, such as clustering or classification, as it allows researchers to focus on the most informative aspects of the data.

The Kaiser-Meyer-Olkin (KMO) measure of sampling adequacy, which stands at 0.911, further supports the appropriateness of conducting PCA on this dataset. A KMO value above 0.6 is generally considered acceptable, indicating that the sample size is sufficient for the analysis. Additionally, Bartlett’s test of sphericity yields a chi-square value of 616.912 with a p-value less than 0.0001, confirming that the correlation matrix is significantly different from an identity matrix. This result reinforces the validity of the PCA, as it indicates that there are meaningful correlations among the variables.

The analysis also provides insights into the contributions of each variable to the principal components. By examining the factor loadings, researchers can identify which variables are most influential in shaping the principal components. This information is crucial for understanding the data structure and can guide further investigations into the genetic diversity represented in the ISSR analysis.

Moreover, the correlation matrix reveals the relationships between the variables, highlighting significant correlations that can inform researchers about the interactions among different genetic markers. The results from the PCA can be visualized through biplots, which illustrate the relationships between observations and variables in the reduced dimensional space, making it easier to interpret the data.

In summary, the PCA conducted on the ISSR dataset not only simplifies the data but also enhances the interpretability of the relationships among variables. The significant variance explained by the principal components, along with the supportive statistical measures, underscores the effectiveness of PCA as a tool for analyzing complex datasets in genetic research. The findings from this analysis can serve as a foundation for more advanced studies, enabling researchers to explore genetic patterns and relationships with greater clarity and precision, ultimately contributing to a deeper understanding of genetic diversity and its implications in various biological contexts.

The PCA conducted on both the RAPD and ISSR datasets provides significant insights into the relationships among the variables involved in genetic diversity studies. The RAPD dataset consists of 60 observations across 13 variables, while the ISSR dataset includes 73 observations with the same number of variables. In both analyses, PCA effectively reduces dimensionality while retaining a substantial amount of variance, with the first principal component explaining 54.32% of the total variance in the RAPD dataset and 56.28% in the ISSR dataset. The cumulative variance explained by the first two components in the RAPD analysis reaches 63.69%, while the first four components in the ISSR analysis account for 77.36% of the total variance.

The Kaiser-Meyer-Olkin (KMO) measure indicates that the samples are adequate for PCA, with values of 0.846 for RAPD and 0.911 for ISSR, both exceeding the acceptable threshold of 0.6. Additionally, Bartlett’s test of sphericity confirms that the correlation matrices for both datasets are significantly different from identity matrices, with p-values less than 0.0001. These results reinforce the validity of PCA, indicating meaningful correlations among the variables.

The analyses also provide insights into the contributions of each variable to the principal components. By examining the factor loadings, researchers can identify which variables are most influential in shaping the principal components for both datasets. This information is crucial for understanding the data structure and can guide further investigations into the genetic diversity represented in both RAPD and ISSR analyses.

Moreover, the correlation matrices reveal the relationships between the variables, highlighting significant correlations that inform researchers about the interactions among different genetic markers. The results from the PCA can be visualized through biplots, illustrating the relationships between observations and variables in the reduced dimensional space, making it easier to interpret the data.

In summary, the PCA conducted on both the RAPD and ISSR datasets not only simplifies the data but also enhances the interpretability of the relationships among variables. The significant variance explained by the principal components, along with the supportive statistical measures, underscores the effectiveness of PCA as a tool for analyzing complex datasets in genetic research. The findings from these analyses can serve as a foundation for more advanced studies, enabling researchers to explore genetic patterns and relationships with greater clarity and precision, ultimately contributing to a deeper understanding of genetic diversity and its implications in various biological contexts.

Genetic relationships among a group of genotypes through molecular and phenotypic data representation may be performed utilizing multivariate techniques, which may intensify the information of many alleles and loci into several copied variables [87]. It is reported that PCA in combination with cluster analysis is a useful tool to extract maximum information from molecular marker data, if the first two or three components explain > 25% of the original variation [88]. Additionally, Gelotar et al. [89] indicated that the results of the PCA were comparable to those obtained with cluster analysis. The grouping of the genotypes in the PCA corresponded with the cluster analysis with some minor differences. Similarly, minor discrepancies were also reported by Oduwaye et al. [90] in ISSR analysis. Previous studies of Kapuria et al. [91] and Chaudhari et al. [92], provided that the PCA analysis has been used for the diversity analysis using RAPD and SSR markers, respectively, which supported the cluster analysis.

### Mantel test analysis

Mantel test was conducted in the document focuses on the relationship between two genetic distance matrices derived from different molecular markers, RAPD and ISSR. These molecular techniques are widely used in genetic studies to assess genetic diversity and relationships among populations.

**Fig. 7:**
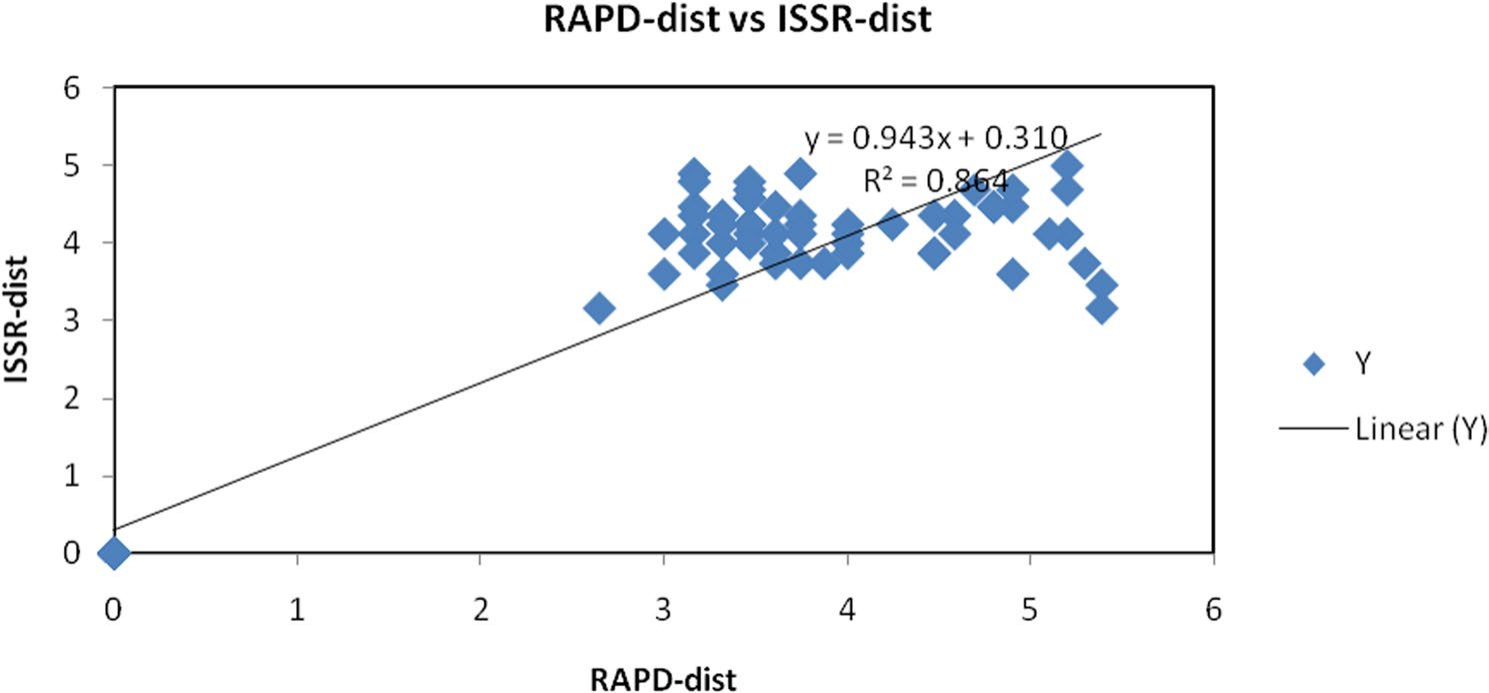
Mantel test showing the correlation between the genetic distance obtained from RAPD, ISSR and cumulative analysis among *Pelargonium graveolens* at different treatment

The linear regression equation provided is: y = 0.9437x + 0.3105. This equation indicates a strong correlation between the distances calculated from the two methods. The slope of the line, (0.9437), suggests that for every unit increase in the ISSR distance, the RAPD distance increases by approximately 0.9437 units (Fig. 7) This close-to-one slope indicates that both methods yield similar results in terms of genetic distance.

The coefficient of determination, denoted as (R^2^ = 0.8647), suggests that approximately 86.47% of the variance in the RAPD distance can be explained by the ISSR distance. This high R^2^ value is indicative of a robust relationship, implying that the two molecular techniques yield similar insights into the genetic diversity among the samples studied. Such a strong correlation is crucial for researchers as it validates the use of these markers in genetic studies.

The scatter plot data points, which range from 0 to 6 on both axes, further illustrate the relationship between the two distance measures. The linear trend observed in the plot signifies that as the genetic distance measured by ISSR increases, the genetic distance measured by RAPD also tends to increase. This reinforces the idea that both methods are capturing similar genetic variations within the samples, which is essential for understanding population structure and dynamics.

Moreover, the intercept of the regression line, (0.3105), suggests that even when the ISSR distance is zero, there is a baseline level of genetic distance observed in the RAPD analysis. This could indicate inherent differences in the genetic makeup of the samples that are not captured by the ISSR method alone. Such findings highlight the importance of considering multiple molecular markers to gain a comprehensive understanding of genetic diversity.

In conclusion, the analysis demonstrates a significant correlation between the genetic distances derived from ISSR and RAPD markers, highlighting the utility of both methods in genetic studies. The findings suggest that researchers can confidently use either technique to assess genetic diversity, as they provide complementary insights into the genetic structure of populations. This analysis not only contributes to the understanding of genetic relationships but also emphasizes the importance of using multiple molecular markers in genetic research to obtain a comprehensive view of genetic diversity. By integrating results from both ISSR and RAPD, researchers can enhance the accuracy of their genetic assessments and better inform conservation strategies and breeding programs (Fig. 7).

Similar observation of positive correlation between the genetic matrices of RAPD, ISSR and SCoT markers was earlier reported [93]. Positive correlations were observed between the genetic matrices of RAPD, ISSR and SCoT with pooled RAPD-ISSR-SCoT matrix, suggesting the effectiveness of RAPD, ISSR and SCoT markers in determining genetic polymorphism and somaclonal variation, albeit very low between the in vitro regenerates and the mother plant. In addition PicoMendoza et al. [94] found that, a null correlation between geographic and genetic distances among populations (R^2^ = 0.007; *P* = 0.02) was detected by the Mantel test, indicating no significant isolation by distance. Nagori et al. [95] demonstrated that the both ISSR and RAPD markers are suitable for characterization and assessment of genetic diversity in *A. squamosa*. The data provides an opportunity for improvement and genetic conservation program of tree as well as in making rational base decision regarding prioritizing population which requires conservation. Furthermore, Mir et al. [96] pointed that Pair-wise Mantel test was performed among genetic distance matrices obtained from RAPD and ISSR molecular markers and it showed statistically significant correlation between the two markers (*r* = 0.634 and *P* = 0.02).

## Materials and methods

### Materials

#### Chemicals and reagents

Murashige and Skoog medium, agar, benzyl-ade nine, indole acetic acid were supplied from Duchefa Biochemie (A. Hofmanweg 71, 2031 BH Haarlem, Netherlands), colchicine, dinitroaniline were supplied by Roth Company (Overland Park, KS, United States). The other reagents that were used in each experiment were analytical grade and obtained from (Acmatic For Chemicals & Lab. Equipment Company, Cairo, Egypt).

#### Plant materials

The plant materials were obtained from the Medicinal and Aromatic Plants Research Department and propagated in the biotechnology lab at the Horticulture Research Institute, Agriculture Research Centre, Giza, Egypt.

The current investigation was applied to study the effect of gamma rays and laser irradiation, as well as chemical mutagens (colchicine and dinitroaniline), on produced shoot survival ability in green house.

The culture media was performed in according to Murashige and Skoog [97] (MS). The media were supplemented with BAP (2.5 mg/l), IAA (2 mg/l), sucrose (3% w/v) and agar-agar (0.7%) as a solidified agent, and pH was adjusted to 5.8 prior to autoclaving. Fifty ml of solidified media were poured into heat sterilized 300 ml glass jars and autoclaved at 121 °C for 20 min. Culture jars were incubated at 25 ± 2 °C (relative humidity 80%) with a 16/8 h photoperiod under white fluorescent tubes (photosynthetic photon flux of 40 µmol m^−2^s^−1^). The growing shoots were subcultured every four weeks until the needed numbers of plantlets for the experiment were reached. Branches with seven to eight leaves and 6–8 cm in length were utilized for different treatments.

.

#### Irradiation treatment

The irradiation procedure of geranium (*P. graveolens*) plantlets was done at the National Centre for Radiation Research and Technology (NCRRT), Cairo, Egypt, by ^60^Co (Indian Gamma cell) Ge-4000 A. After 4 weeks from the subculture, the jars with plantlets were irradiated by gamma rays at various doses level; 5, 10, 20, and 40 Gy at a dose rate of 0.43 Gy/min (Table 7) at the time of irradiation process. After γ-irradiation, the shoots were immediately transferred to the fresh same multiplication medium [98].

#### Laser procedure

After four weeks from the subculture, the jars with geranium plantlets were transferred to Petri dishes and exposed to laser He-Ne (wavelength 632.8 nm, power density 30.0 mW, beam diameter 1.0 mm model Griat, U.S.A.) for 5, 10, 15 and 30 min. (Table 7). Laser technique was performed at the National Institute of Laser Enhanced Sciences (NILES), Laser Technology Centre, Cairo University, Giza, Egypt.

#### Chemical mutagenic agent

Colchicine and dinitroaniline solutions (Table 7) were performed by mixing them and dissolved according to the treatment concentrations [99], with MS media at concentrations of 5.0 and 10.0 mg/l for twenty- four hours. Mother plant treatment was performed utilizing MS medium (not including colchicine or dinitroaniline). Auxiliary shoots that were immersed in colchicine and dinitroaniline solutions were planted in multiplication culture media and incubated for 4 weeks. After three subcultures for the above- mentioned treatments, plantlets (10 ± 2 cm) produced from the in vitro rooting phase were with arm (40–50 °C) water was used to remove the agar debris from the young roots, and the plantlets were transferred to the pots containing peat moss: perlite (1:1) and were placed in a growth chamber under relative humidity 70–80%, a 16:8 photoperiod regime and a 23 ± 1 °C and 20 ± 1 °C temperature regime during the day and night, respectively. Acclimatized plantlets were moved to the greenhouse 4 weeks after transfer to soil, and were water irrigated twice a week for four weeks before transplanting. After that acclimatized plantlets were moved to the greenhouse for further grown. At the end of this experiment survival percentage, plant length (cm), number of branches/plant, number of leaves/plant and leaves fresh and dry weights (g/plant) were recorded well as and other samples were taken for further analysis. Each treatment consisted of 10 pots, each containing 6 plantlets; this resulted in a total of 60 plants, which divided them into three replicates, each containing 20 plants. Five plants were randomly selected from each replicate for morphological assessment. The research is planned utilizing a full randomized blocks design with three repeats, at the experimental farm of the Horticulture Research Institute and Biotechnology Laboratory, Horticulture Research Institute, Agriculture Research Centre, Giza, Egypt.

**Table 7:** Codes and sources of the different treatments

Different treatments	
Mother plants	
Gamma rays (Gy)	5
	10
	20
	40
He-Ne Laser (min)	5
	10
	15
	30
Colchicine (mg/l)	5
	10
Dinitroaniline (mg/l)	5
	10

#### Essential oil content of Pelargonium graveolens

The percent of essential oil in fresh leaves was evaluated using the gas chromatography mass-spectroscopy GC-MS analyses achieved by a (Thermo Scientific, Trace GC Ultra/ISQ Single Quadrupole MS Taiwan) [100]. The TG-5MS combined silica capillary column (30 m, 0.251 mm, 0.1 mm film thickness). For GC-MS detection, an electron ionization system with ionization energy of 70 eV was used Helium gas served as the carrier for the GC-MS detection system, which engaged as an electron ionization system with ionizing energy of 70 eV and a constant flow rate of 1.0 ml/min. The temperatures of the injection and MS transfer line were set at 280 °C. The oven temperature was scheduled to begin at 40 °C (hold for 3 min) to increase by 5 °C/min (hold 5 min) until it reached to ending temperature of 280 °C. The relation percentage quantity of every compound was calculated using its average peak area with the total area by managing GC-MS Turbomass software. The National Institute of Standards and Technology (NIST) database, which has over 62,000 patterns, was utilized to clarify the mass spectrum GC-MS of the tested samples. The compounds of the treated treatments were identified by their names, structure, and molecular weight [101].

### Molecular genetics identification using RAPD and ISSR methods

#### DNA extraction

Total genomic DNA was isolated and purified from the frozen leaves of *P. graveolens* different samples using the DNeasy Plant Mini Kit (QIAGEN, Chatsworth, CA). The DNA content was calculated at 260 nm, and the excellence was confirmed by electrophoresis on 1.4% agarose gel.

### RAPD-PCR Analysis

#### Polymerase chain reaction (PCR)

According to Williams et al. [102], a total of twenty random DNA oligonucleotide primers were utilized independeapproximately 0.9437 unitsntly in the PCR mixture. The PCR magnification was carried out in a 25 µl reaction amount including the following: 2.5 µl of dNTPs (2.5mM), 1.5 µl of MgCl_2_ (25mM), 2.5 µl of 10x buffer, 2.0 µl of primer (2.5µM), 2.0 µl of template DNA (50ng/µl), 0.3 µl of Taq polymerase (5U/µl) and 14.7 µl of sterile ddH_2_O. The reaction mixtures were overlaid with a drop of light mineral oil per treatment. Amplification is performed using the (Techni TC-512 PCR System, UK). The reactions were subjected to one cycle at 95 °C for 5 min., followed by 35 cycles at 96 °C for 30 s, 37 °C for 30 s, and 72 °C for 30 s, then the final cycle of 72 °C for 5 min. The PCR results were run at 100 V for one h in 1.5% agarose gel to identify polymorphism among the geranium treatments that were being investigated. Only 6 primers succeeded to generate reproducible polymorphic DNA products. Table (8) lists the base sequence of these DNA primers that produced useful polymorphic bands. The PCR results were run in a 1.5% agarose gel, and fragment sizes are determined with the 100 bp ladder marker (1500, 1000, 900, 800, 700, 600, 500, 400, 300, 200, and 100 bp).

### ISSR-PCR analysis

#### Polymerase chain reaction (PCR)

Ten primers were utilized to conduct ISSR-PCR experiments. Amplification was performed in 25 µl reaction amount including 2.5 µl of dNTPs (2.5mM), 2.5 µl MgCl_2_ (2.5 mM), and 2.5 µl of 10X buffer, 3.0 µl of primer (10 pmol), 3.0 µl of template DNA (25ng/µl), 1.0 µl of Taq polymerase (1U/µl) and 12.5 µl of sterile ddH_2_O. The PCR reactions were programmed for one cycle at 94 °C for 4 min followed by 45 cycles of one min at 94 °C, one minute at 57 °C, and 2 min. at 72 °C, the reactions were finally stored at 72 °C for 10 min. The PCR products were separated on a 1.5% agarose gel, and fragment sizes were evaluated by the 100 bp ladder marker (1500, 1000, 900, 800, 700, 600, 500, 400, 300, 200, and 100 bp).

Six primers only succeeded to generate reproducible polymorphic DNA products. Table 8 listing the base sequences of these DNA primers that generated informative polymorphic bands.

**Table 8:** The primer names and their nucleotide sequences of RAPD and ISSR procedures used in the study

RAPD Primers		ISSR Primers	
Primer Name	Sequence	Primer Name	Sequence
OP-A01	5`CAGGCCCTTC3`	14 A	5`CTCTCTCTCTCTCTCTTG3`
OP-A09	5`GGGTAACGCC3`	44B	5`CTC TCT CTC TCT CTC TGC3`
OP-B11	5`GTAGACCCGT3`	HB-9	5`GTG TGT GTG TGT GC 3`
OP-C04	5`CCGCATCTAC3`	HB-11	5`GTG TGT GTG TGT TGT CC3`
OP-C09	5`CTCACCGTCC3`	HB-12	5`CAC CACCAC GC 3`
OP-Q18	5`AGGCTGGGTG3`	HB-15	5`GTG GTGGTG GC 3`

### Statistical analyses

The experimental design was randomized block design with three replicates as described by Sndecor and Cochran [103] and L.S.D. at (0.05% level) for comparing the averages of different samples. The achieved PCR products were electrophoresed by agarose gel electrophoresis as described by Williams et al. [102]. The obtained DNA bands by each primer counted and their molecular sizes compared with those of the DNA markers. The bands scored from DNA profiles generated by each primer were pooled together. After that the presence or absence of every DNA band was treated as a binary character in a data matrix (coded 1 and 0, respectively) to calculate genetic similarity and to construct dendrogram tree among the studied *Pelargonium graveolens* samples. Calculation accomplished by Dice similarity coefficients [104]. All data set performed by SPSS (version. 14.0) program. The PCA was crried out as described by Abou-Serra et al. [105] and Mantel test as described by Mantel and Valaned [106].

## Conclusion

The current research clarified the morphological, biochemical, and genetic properties of *Pelargonium graveolens* mother plant and it’s induced plants from different treatments to determine the best treatment induced by physical and chemical mutagens. The treatments 5 and 10 Gy are promising for inducing mutant plants, which had the highest survival percentage. In this concern, the greatest plant length was found in 40 Gy treatment, while 10 mg/l colchicine had the maximum number of branches. Moreover, 5 mg/l colchicine had the greatest volatile oil percentage. In addition, He-Ne laser 5 min and 10 Gy treatments had the highest number of leaves and the greatest fresh weight respectively. Furthermore, it can be concluded that the molecular analysis of RAPDs and ISSRs markers were particularly helpful for understanding the genetics of *P. graveolens* and its induced treatment. These analyses also made the markers as a potent tool for the generating of possible fingerprinting markers. Also, the PCA and mantel test were performed for the molecular data. Finally, the current study suggested the using both, the application of gamma-rays, laser light and chemical mutagens had a valuable effect on vegetative development characters and oil production of *P. graveolens*, the treated samples also showed improved essential oils and its components when compared to untreated mother plant. Also, molecular and physiological studies could supply deeper approaching into the underlying mechanisms.

The combined treatments of physical and chemical mutagens are of perceptible awareness to a mutation breeder with an intention of enhancing mutation spectrum and frequency, thereby maximizing labors to gain affirmative results.

## Supplementary Information

Supplementary Material 1.

Supplementary Material 2.

Supplementary Material 3.

Supplementary Material 4.

Supplementary Material 5.

Supplementary Material 6.

Supplementary Material 7.

Supplementary Material 8.

Supplementary Material 9.

Supplementary Material 10.

Supplementary Material 11.

Supplementary Material 12.

Supplementary Material 13.

## Data Availability

The data sets used and/or analyzed during the current study available from the corresponding author on reasonable request.

## Abbreviations

He-Ne laser: Helium-Neon laser
GC/MS: Gas Chromatography Mass-Spectrometry
PCR: Polymerase Chain Reaction
RAPD: Randam Amplified Polymorphic DNA
ISSR: Inter Simple Sequence Repeat
SCoT: Start Codon Targeted
miRNA: micro Ribonucleic Acid
DNA: Deoxyribonucleic Acid
SSR: Simple Sequence Repeats
SRAP: Sequence-Related Amplified Polymorphism
TAF: Total Amplified Fragments,
MF: Monomporphic Fragments,
PF: Polymorphic Fragments,
SM: Specific Markers
UPGMA: Unweighted Pair Group Method using Arithmetic averages
PCA: Principal Component Analysis (PCA)
