# Supplementary figures and images for: Evaluation of in vitro-geranium (Pelargonium graveolens) plants affected by irradiation and chemical mutagens
Source: BMC Plant Biol. 2025 Oct 23;25:1447. doi: 10.1186/s12870-025-07170-w (PMC12548295; doi:10.1186/s12870-025-07170-w)

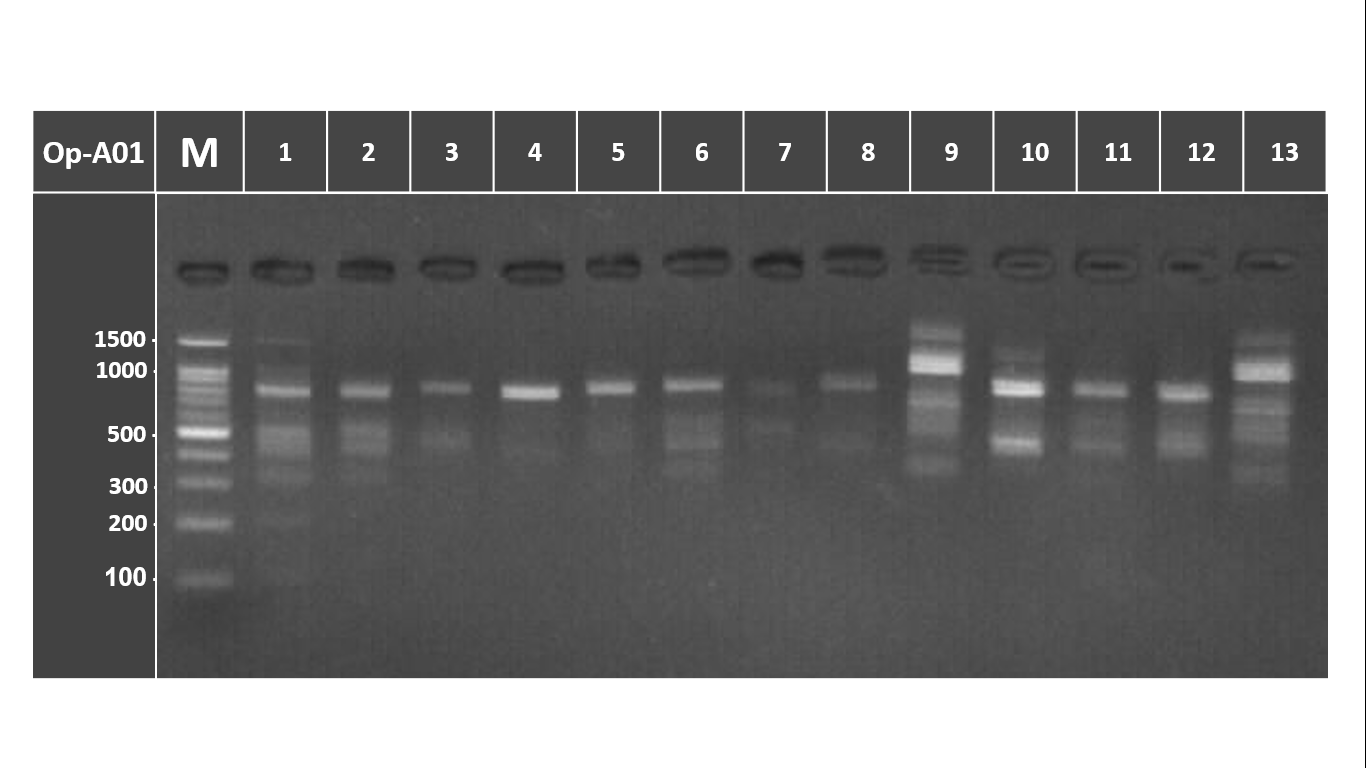

Supplement: Supplementary file 1 — Supplementary Material 1. [file 12870_2025_7170_MOESM1_ESM.tif]

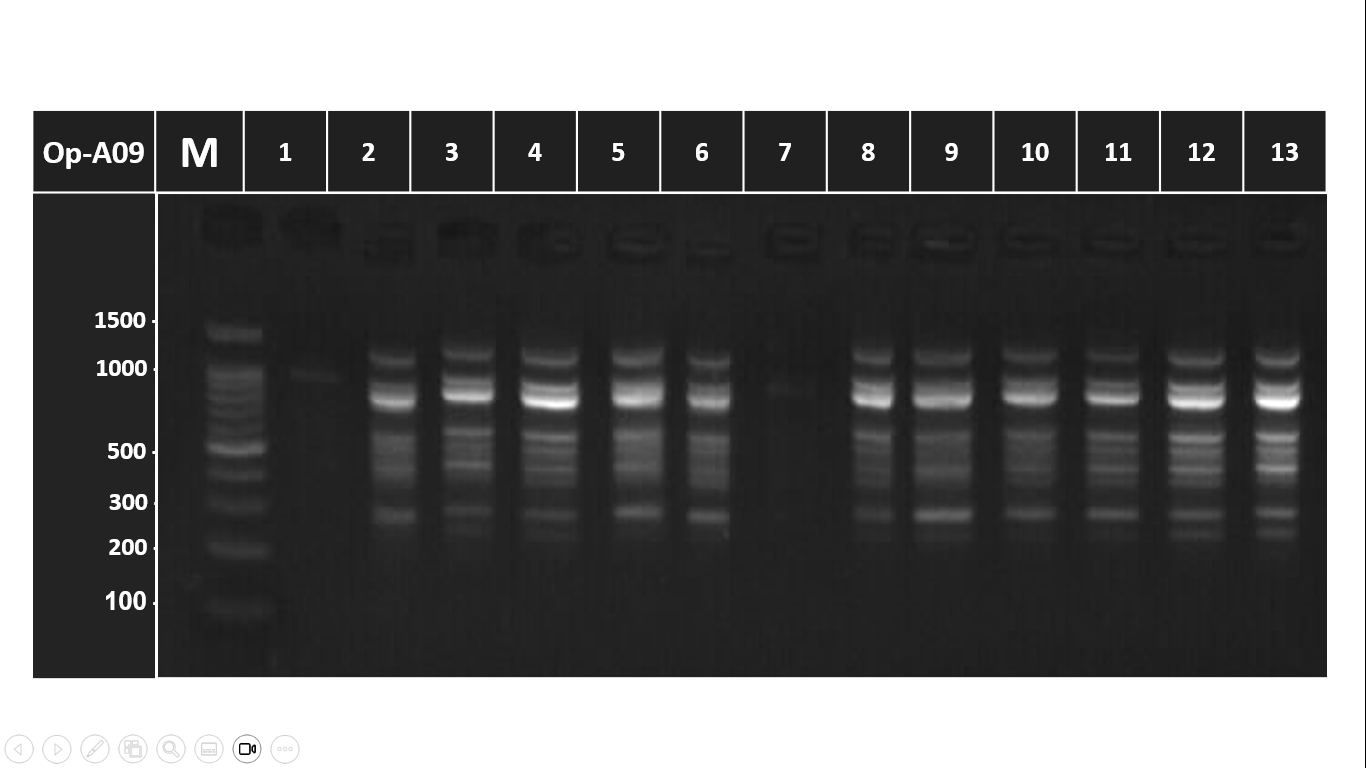

Supplement: Supplementary file 2 — Supplementary Material 2. [file 12870_2025_7170_MOESM2_ESM.tif]

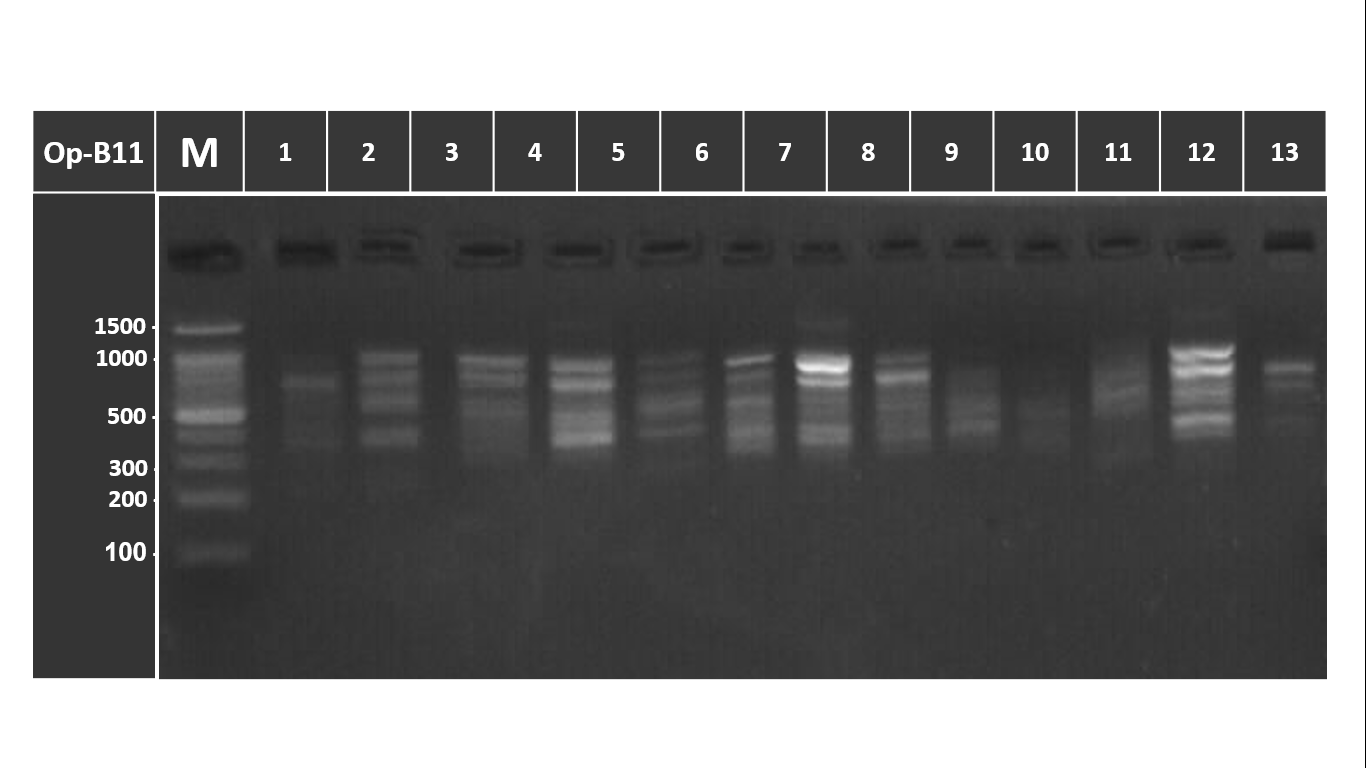

Supplement: Supplementary file 3 — Supplementary Material 3. [file 12870_2025_7170_MOESM3_ESM.tif]

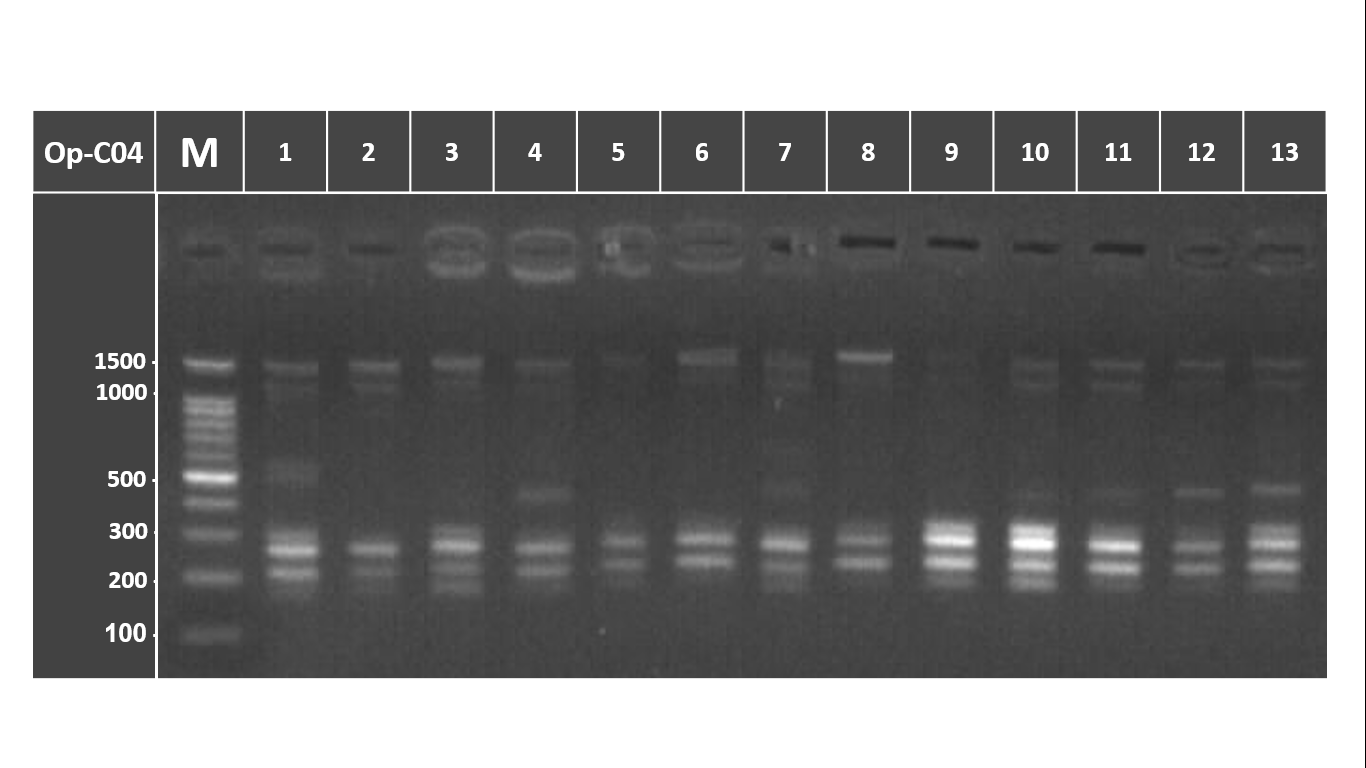

Supplement: Supplementary file 4 — Supplementary Material 4. [file 12870_2025_7170_MOESM4_ESM.tif]

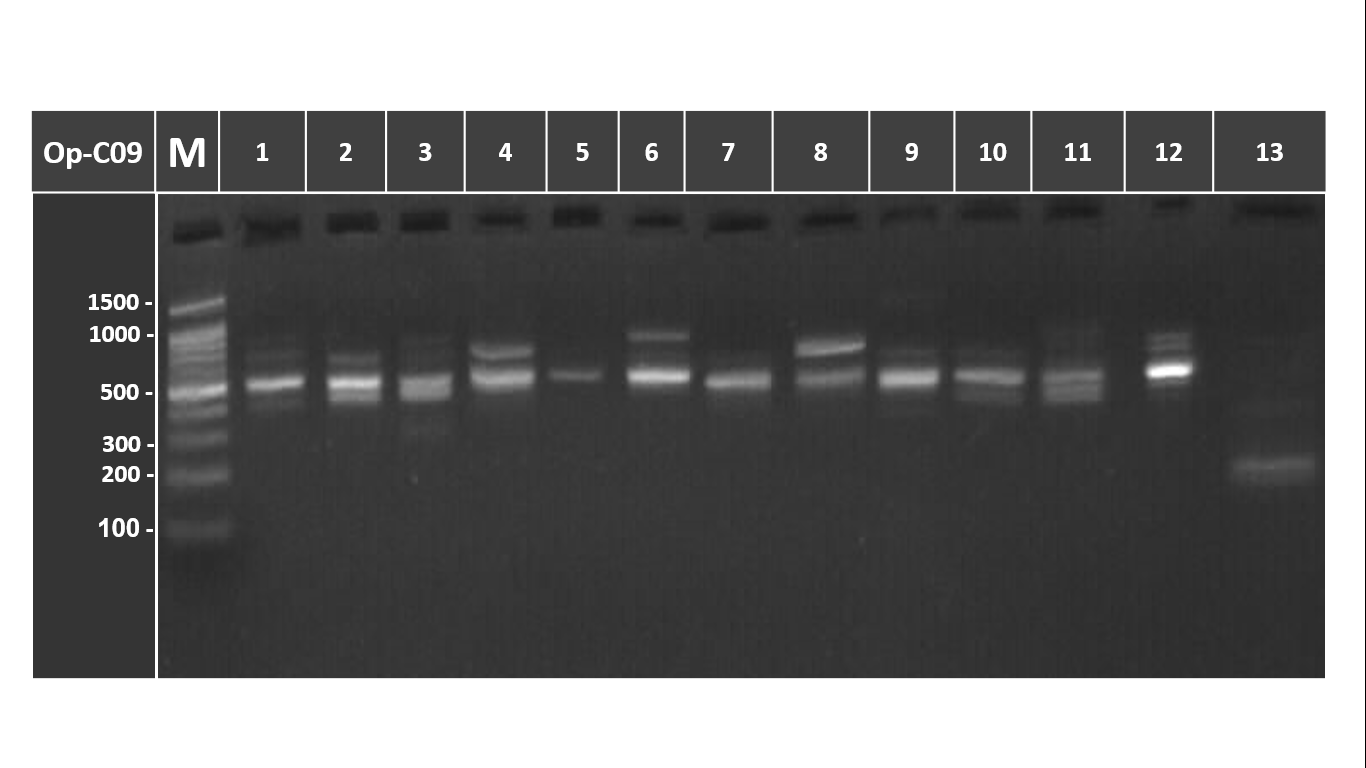

Supplement: Supplementary file 5 — Supplementary Material 5. [file 12870_2025_7170_MOESM5_ESM.tif]

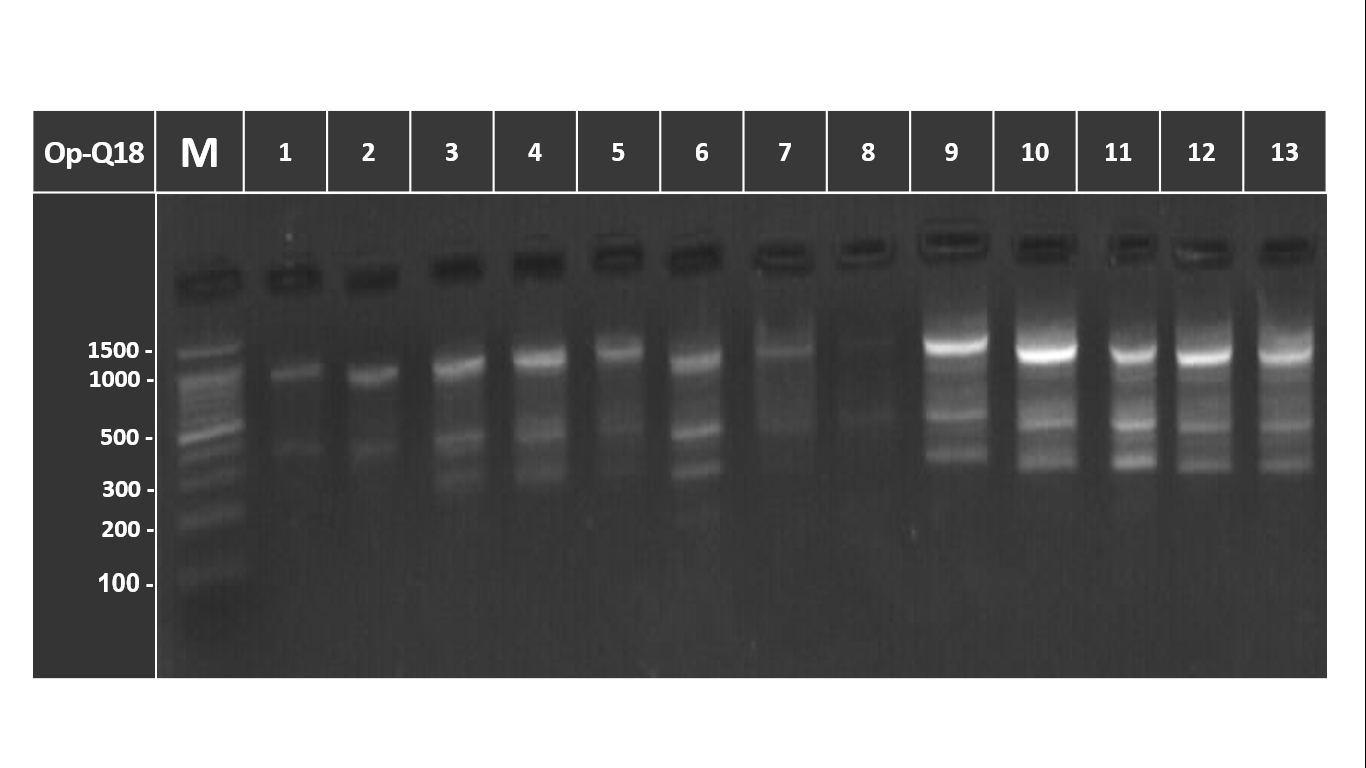

Supplement: Supplementary file 6 — Supplementary Material 6. [file 12870_2025_7170_MOESM6_ESM.tif]

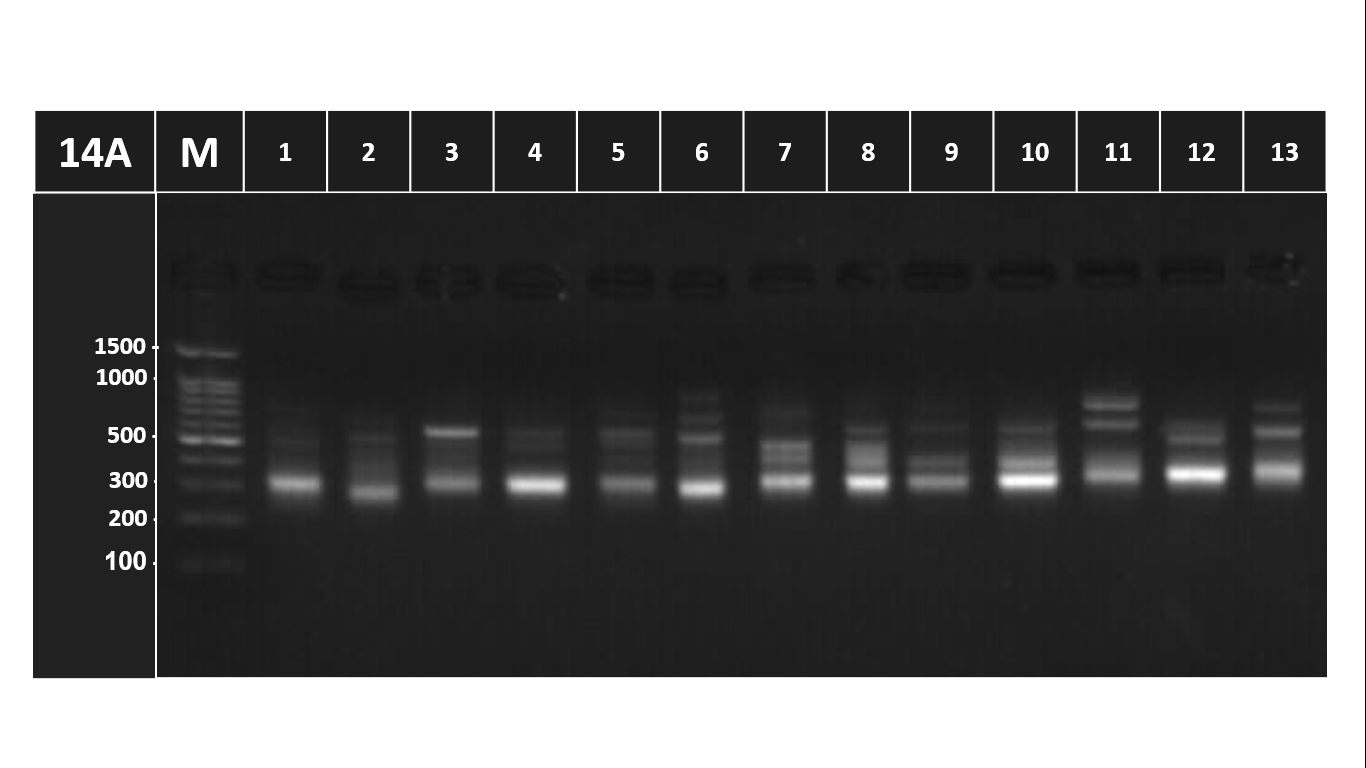

Supplement: Supplementary file 7 — Supplementary Material 7. [file 12870_2025_7170_MOESM7_ESM.tif]

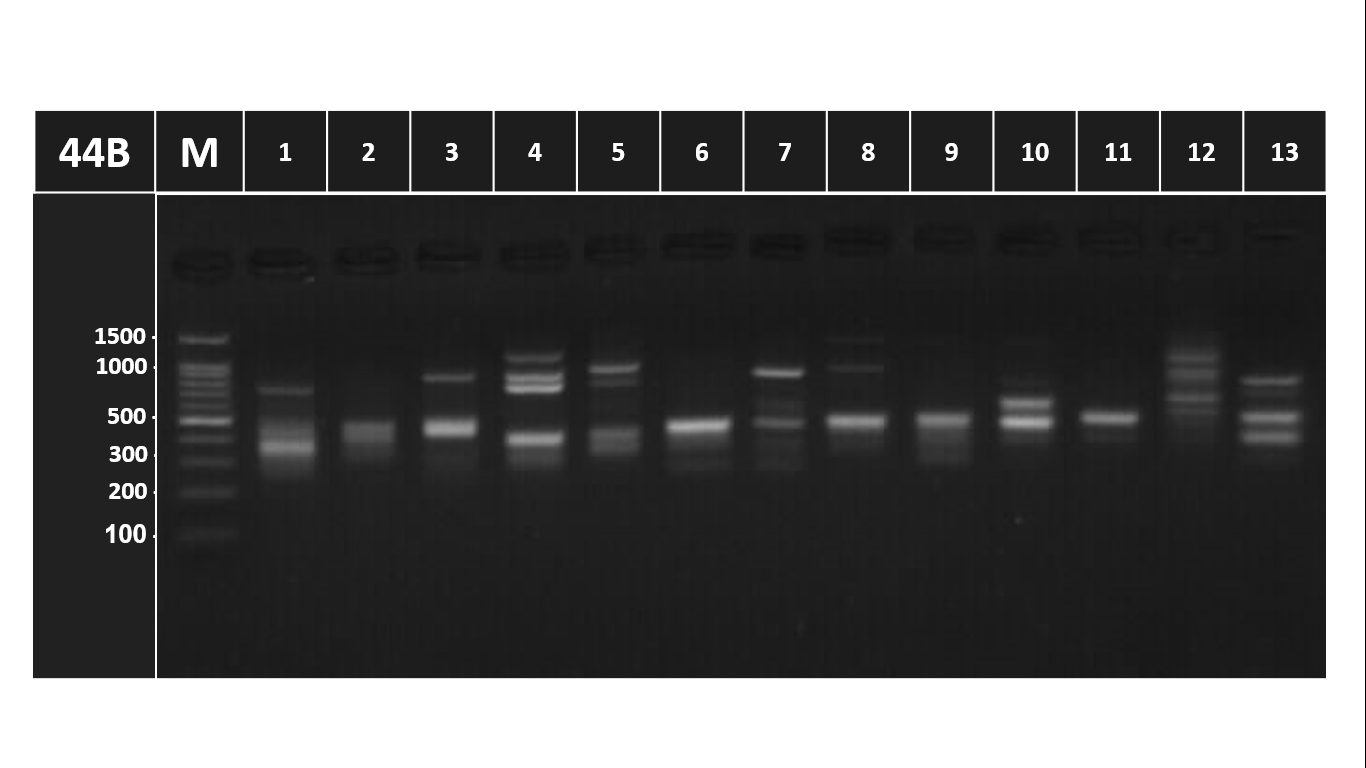

Supplement: Supplementary file 8 — Supplementary Material 8. [file 12870_2025_7170_MOESM8_ESM.tif]

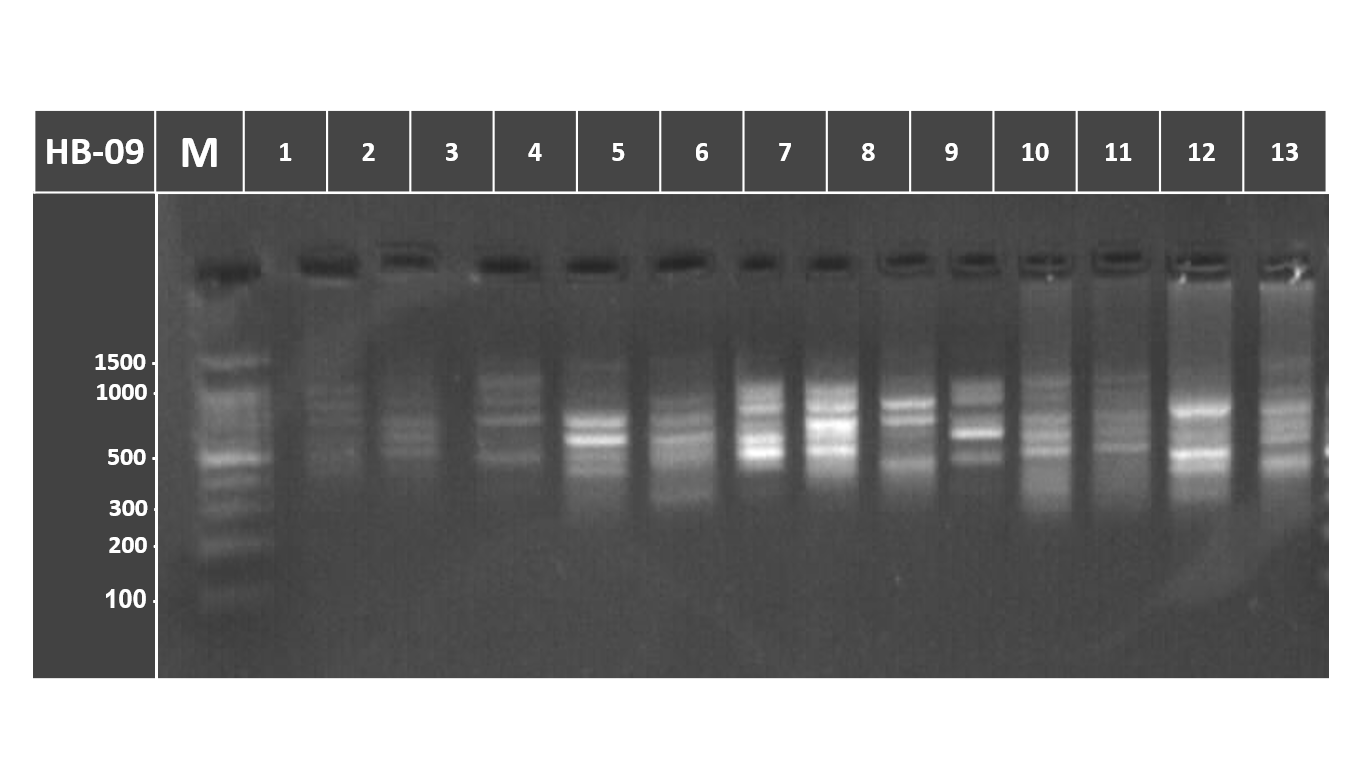

Supplement: Supplementary file 9 — Supplementary Material 9. [file 12870_2025_7170_MOESM9_ESM.tif]

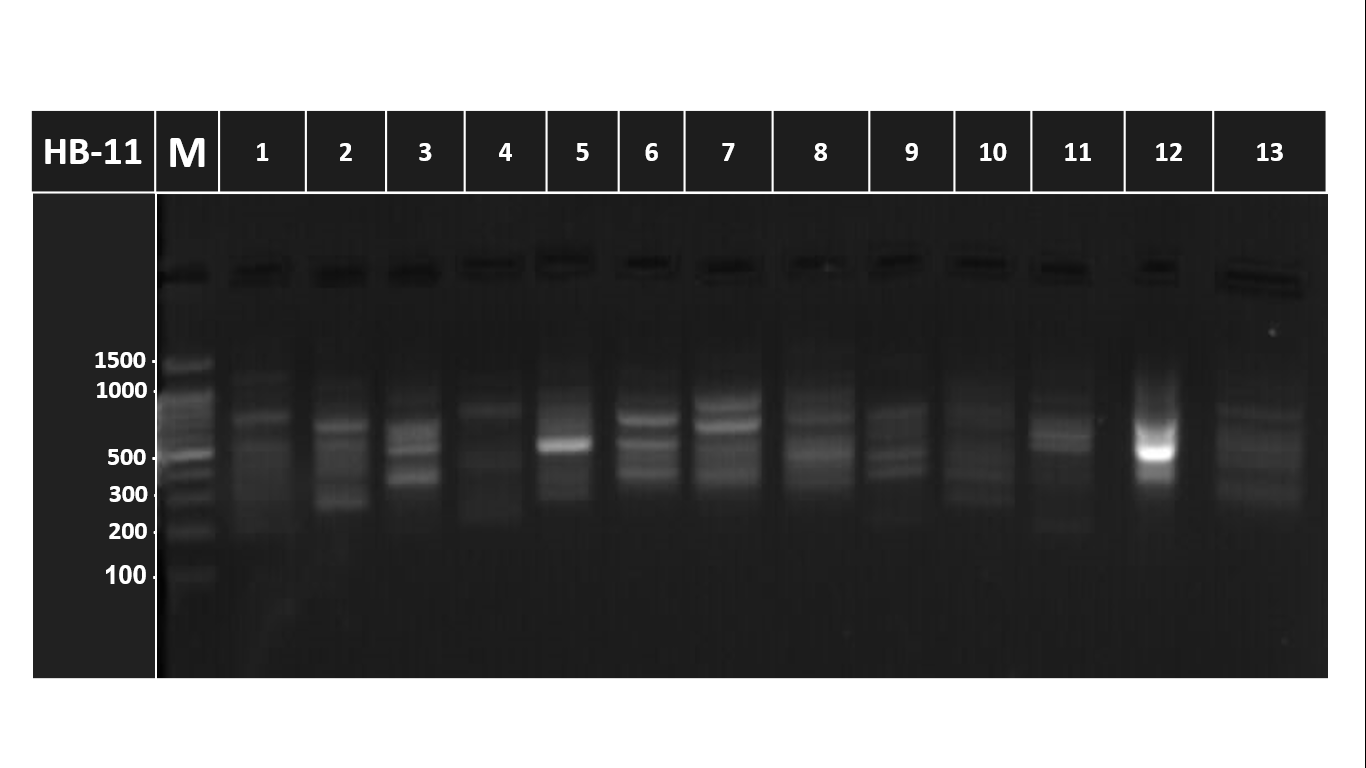

Supplement: Supplementary file 10 — Supplementary Material 10. [file 12870_2025_7170_MOESM10_ESM.tif]

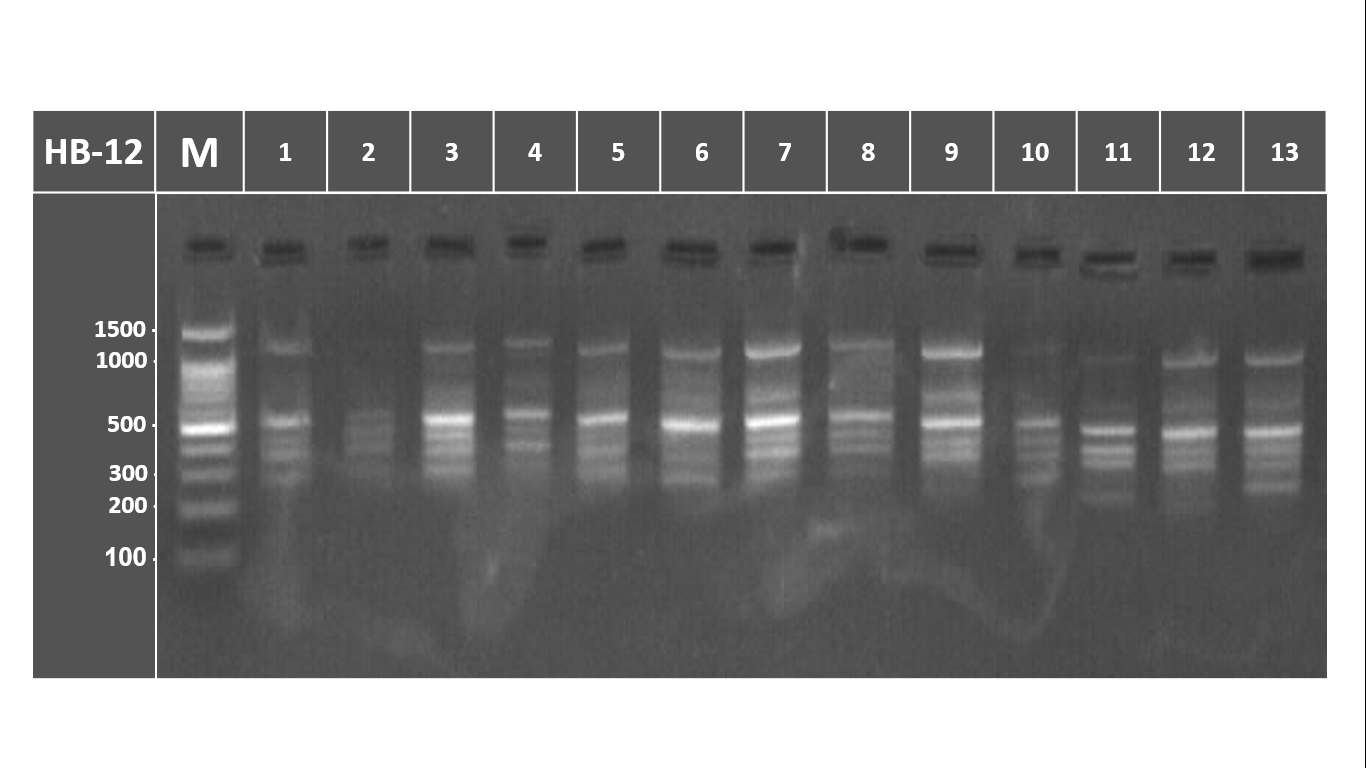

Supplement: Supplementary file 11 — Supplementary Material 11. [file 12870_2025_7170_MOESM11_ESM.tif]

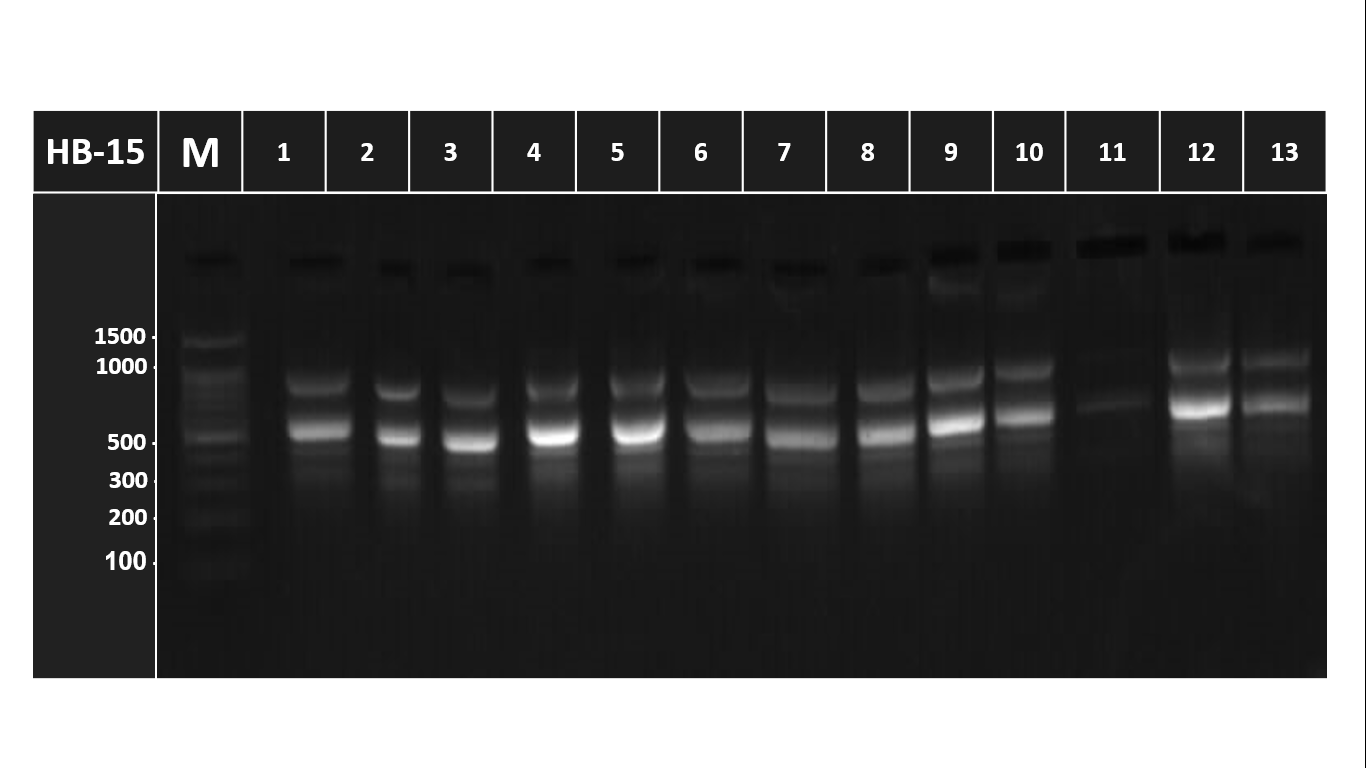

Supplement: Supplementary file 12 — Supplementary Material 12. [file 12870_2025_7170_MOESM12_ESM.tif]
