# Supplementary material for: Evaluation of in vitro-geranium (Pelargonium graveolens) plants affected by irradiation and chemical mutagens
Source: BMC Plant Biol. 2025 Oct 23;25:1447. doi: 10.1186/s12870-025-07170-w (PMC12548295; doi:10.1186/s12870-025-07170-w)
Supplement: Supplementary file 13 — Supplementary Material 13. [file 12870_2025_7170_MOESM13_ESM.doc]

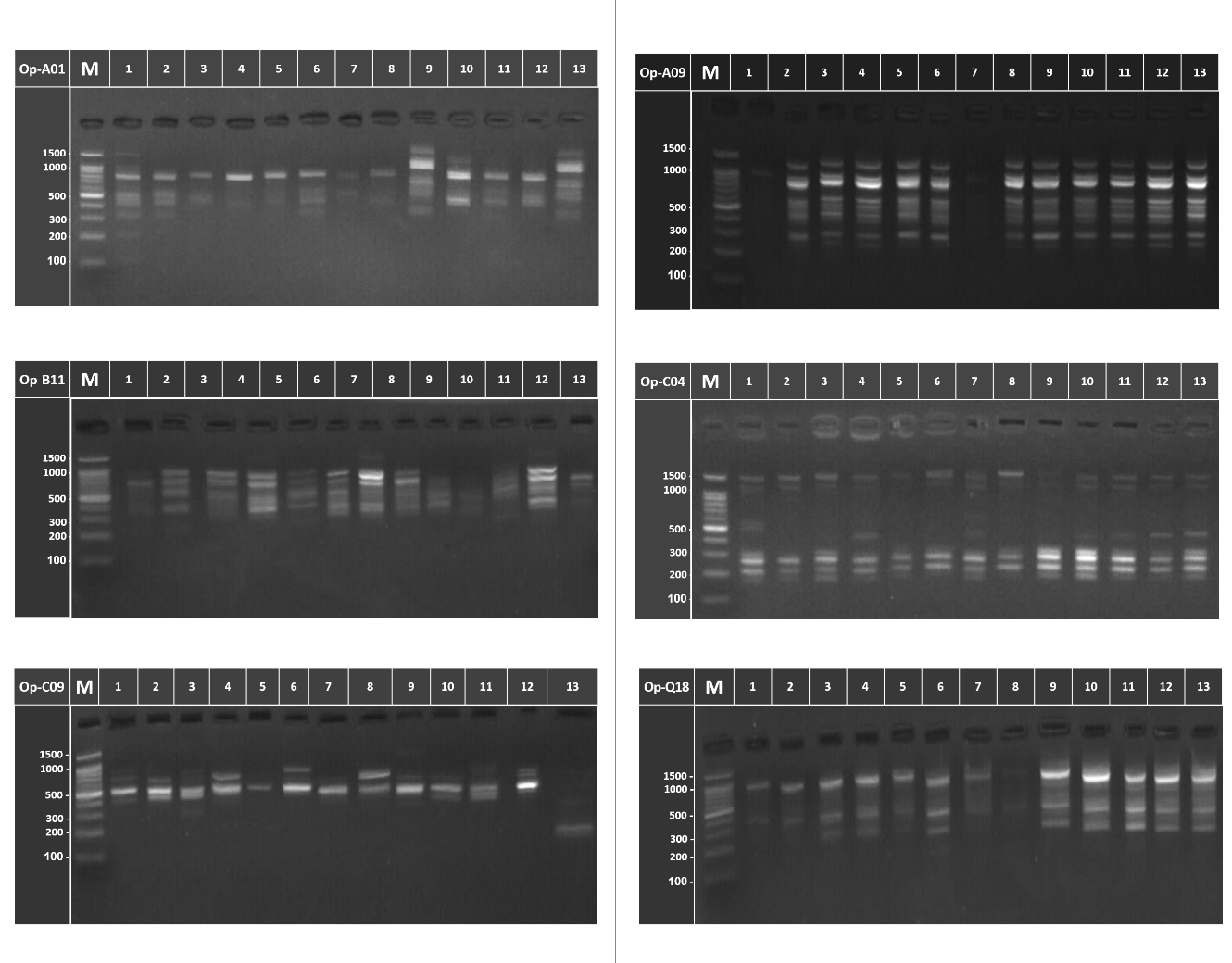


**Fig. 1** RAPD-PCR analysis of the mother plants of *P. graveolens* and its induced plants from different treatments *in vitro* (1=Mother plant, 2= 5 Gy, 3= 10 G 4= 20 Gy, 5= 40 Gy, 6= He-Ne laser 5 min, 7=He-Ne laser 10 min, 8=He-Ne laser 15 min, 9=He-Ne laser 30 min, 10= 5 mg/l colchicine, 11=10 mg/l colchicine, 12= 5 mg/l dinitroaniline, 13=10 mg/l dinitroaniline).


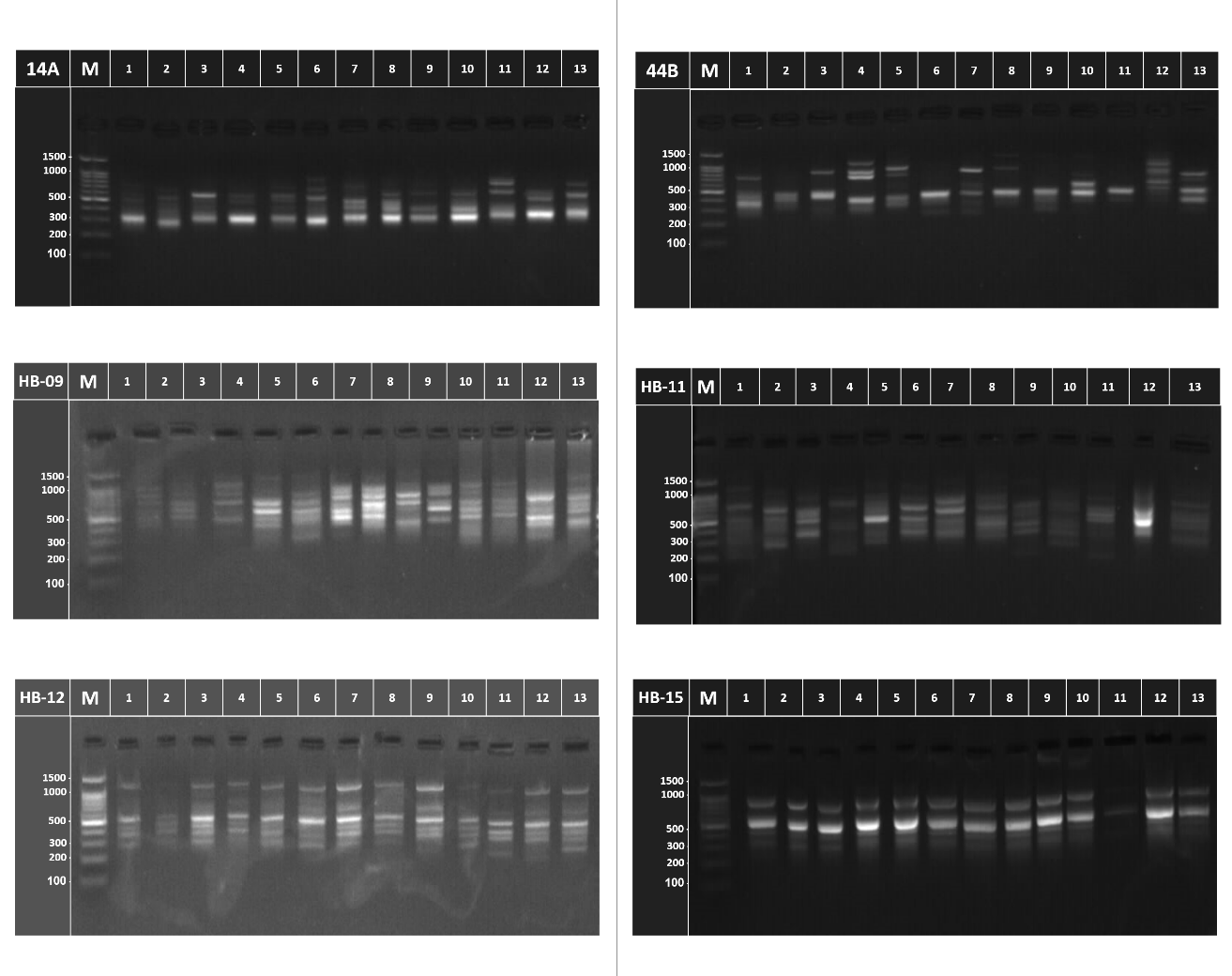


**Fig. 2** ISSR-PCR analysis of the mother plants of *P. graveolens* and its induced plantsfrom different treatments (1=Mother plant, 2= 5 Gy, 3= 10 G 4= 20 Gy, 5= 40 Gy, 6- He-Ne laser 5 min, 7=He-Ne laser 10 min, 8=He-Ne laser 15 min, 9=He-Ne laser 30 min, 10=5 mg/l colchicine, 11=10 mg/l colchicine, 12= 5 mg/l dinitroaniline, 13=10 mg/l dinitroaniline)
